# Nanoscale Characterization of Nanomaterial-Based Systems: Mechanisms, Experimental Methods, and Challenges in Probing Corrosion, Mechanical, and Tribological Properties

**DOI:** 10.3390/nano15231824

**Published:** 2025-12-02

**Authors:** Md Ashraful Hoque, Chun-Wei Yao

**Affiliations:** Department of Mechanical Engineering, Lamar University, Beaumont, TX 77710, USA; mhoque4@lamar.edu

**Keywords:** nanomaterial, nanomaterial-based systems, corrosion, mechanical, tribological, in situ microscopy, nanoscale characterization

## Abstract

Nanomaterial-based systems (NBS) have emerged as transformative elements in advanced surface engineering, offering superior corrosion resistance, mechanical strength, and tribological resilience governed by unique phenomena inherent to the nanoscale. However, bridging the knowledge gap between these enhanced physicochemical properties and the metrological tools required to quantify them remains a critical challenge. This review provides a comprehensive examination of the fundamental mechanisms, state-of-the-art experimental techniques, and computational strategies employed to probe NBS behavior. The article first elucidates the core mechanisms driving performance, including passive barrier formation, stimuli-responsive active corrosion inhibition, grain boundary strengthening, and the formation of protective tribo-films by 2D nanomaterial-based systems. Subsequently, the article evaluates the transition from conventional macroscopic testing to high-resolution in situ characterization, highlighting the capabilities of High-Speed Atomic Force Microscopy (HS-AFM), Liquid Cell Transmission Electron Microscopy (LC-TEM), and nanoindentation in visualizing dynamic defect evolution and measuring localized mechanical responses. Furthermore, the indispensable role of computational materials science—specifically Molecular Dynamics (MD) and Machine Learning (ML)—in predictive modeling and elucidating atomic-scale interactions is discussed. Finally, persistent challenges regarding substrate interference, sample heterogeneity, and instrumentation limits are addressed, concluding with a perspective on future research directions focused on standardization, operando testing, and the development of AI-driven “Digital Twins” for accelerated testing and material optimization.

## 1. Introduction

### 1.1. Overview of Nanomaterial-Based Systems

Nanomaterial-based System (NBS) refers to an integrated assembly where nanomaterials are combined with other components or structures to create a functional system, rather than existing in isolation. This system-level organization often enables enhanced or novel properties that arise from interactions between the nanomaterials and their environment or other materials within the system [1,2]. In contrast, a “simple” nanomaterial typically denotes a single type of nanoparticle or nanostructure without such complex integration. Nanomaterials include particles, tubes, sheets, and other structures with nanoscale dimensions. They can be classified by their shape (0D, 1D, 2D, 3D), composition (carbon-based, metal-based, organic, hybrid), and structure (crystalline, amorphous, heterogeneous) [3,4,5]. The critical dimensional threshold that defines nanomaterials generally lies between 1 and 100 nanometers, where materials exhibit unique size-dependent properties such as altered optical, electronic, or chemical behaviors not seen in bulk materials [6,7]. These nanoscale dimensions trigger quantum effects and increased surface area-to-volume ratios, which are fundamental to the distinct properties exploited in NBS [1,2]. Nanomaterial-based systems are commonly classified based on their dimensionality, composition, morphology, and origin. Dimensionality classification includes zero-dimensional (0D) nanoparticles, one-dimensional (1D) nanorods or nanowires, two-dimensional (2D) atomically thin layers, and three-dimensional (3D) nanostructures, each with distinct physical and chemical properties [3,8]. Composition-wise, nanomaterials are grouped into carbon-based (e.g., carbon nanotubes, graphene, fullerenes), metal-based, polymeric, ceramic, and lipid-based types, with carbon-based nanomaterials being notable for their unique mechanical and electrical properties [9,10]. Morphological classification considers shape and size, such as nanospheres, nanofibers, and nanotubes, which influence their optical and catalytic behaviors [9,11]. Origin-based classification distinguishes natural, incidental, engineered, and bioinspired nanomaterials, reflecting their synthesis routes and applications [3]. Nanomaterial-based systems have diverse applications across biomedical, energy, environmental, and industrial fields due to their unique physicochemical properties. In biomedicine, they are widely used for targeted drug delivery to improve therapeutic efficacy and reduce side effects, as well as in bioimaging, biosensing, tissue engineering, and antimicrobial treatments [9,12]. Nanomaterials also enhance energy storage systems, such as batteries and capacitors, by improving conductivity and surface area, contributing to more efficient and sustainable energy solutions [13,14]. Environmental and agricultural applications include water and soil purification, nano-fertilization, pest control, and food quality enhancement, often leveraging green synthesis methods for sustainability [15]. Additionally, nanomaterials are integral to the development of sensitive nano-sensors for healthcare, environmental monitoring, and security, benefiting from their high surface area and biocompatibility [16]. Nanomaterials are increasingly used in surface engineering—such as coatings, thin films, and multilayered films—to significantly improve corrosion resistance, mechanical strength, and tribological properties at nanoscale. Integrating nanomaterials into coating or protective systems creates more durable, protective, and multifunctional surfaces for metals and other materials [17,18,19,20,21]. Nanomaterials like graphene, MXenes, and hexagonal boron nitride act as barriers, blocking corrosive agents and reducing material degradation. It also increases the hardness, wear resistance, and durability of coatings [17,18]. Nanocomposite coatings with nanomaterials such as carbon nitride (C_3_N_4_) and ZnO-doped C_3_N_4_ nano-capsules improve corrosion protection by filling microdefects and pores in the coating matrix, preventing localized corrosion. ZnO-doped C_3_N_4_ nano-capsules in NiP coatings significantly improve microhardness and wear resistance, especially after heat treatment [20].

### 1.2. Significance of Studying Corrosion, Mechanical, and Tribological Properties of Nanomaterial-Based Systems at Nanoscale

Nanomaterials are playing an increasingly significant role in combating corrosion, particularly through their application in many advanced nanomaterial-based protective systems like coatings, thin films, nano-coatings, and superhydrophobic coatings or surfaces [22,23,24,25,26,27,28,29,30]. These materials, often featuring dimensions below 100 nm, offer unique chemical and physical properties not found in larger-scale materials [27]. As an example, the use of nanoparticles in superhydrophobic coatings enables the formation of hierarchical micro-/nanostructures that trap air to create a cushion that reduces the contact area between water droplets and the surface, thereby enhancing anti-corrosion performance [24,25,29]. Nanocomposite material, consisting of a matrix with dispersed nanofillers, fills voids and blocks corrosive species from diffusing to the substrate surface, leading to lower porosity and cracking potential [26,28]. Nanoparticles can function as corrosion inhibitors by adsorbing at the metal–electrolyte interface to form a protective layer. Their high surface area-to-volume ratio can maximize targeted distribution and efficiency [22]. Some nano-coatings leverage nano-crystallinity, where a smaller grain size can contribute to improved corrosion resistance [23,27]. For example, a nanostructured Ni-Fe alloy coating with a smaller crystallite size exhibited a lower corrosion rate [23]. Smart coatings with core–shell nanofibers containing inhibitors can release protective agents upon environmental triggers, such as a change in pH, providing active corrosion protection [30]. Studying the mechanical and tribological properties of nanomaterial-based systems at the nanoscale is also significant. These materials exhibit exceptional strength, elasticity, and frictional behavior that differ fundamentally from their bulk counterparts due to their unique size, surface effects, and atomic structures [31,32,33,34]. Applications range from enhancing the service life and endurance of machine components, cutting tools, and aerospace applications through coatings like CrN on Ti6Al4V alloy [35], to creating nanoparticle-embedded superhydrophobic coatings that provide not only barrier protection against corrosion but also demonstrate robust mechanical and tribological behavior at nanoscale [29]. However, materials behave distinctly at the nanoscale compared to the macroscale because of the scale effect, which leads to significant alterations in surface roughness, contact characteristics, hardness, and modulus of elasticity [31]. The incorporation of nanomaterials offers solutions to mitigate these issues by improving nanomechanical and nanotribological properties. In case of Si addition, CoCrNi nanopillars significantly enhance strength and stability under compression by influencing dislocation movement and twin formation [36]. Carbon nanotube (CNT) arrayed nanostructures act as effective crack arrestors, substantially improving interlaminar fracture toughness through mechanisms like interlocking, pull-out, nano-bridging, and network formation [37]. Specific coatings demonstrate superior performance; CrN coatings on Ti6Al4V alloys exhibited higher hardness, modulus of elasticity, and notably better adhesive strength and tribological performance than TiN and TiB_2_ [35].

Characterizing the intricate corrosion, mechanical and tribological behaviors necessitates the use of advanced experimental techniques capable of providing high spatial resolution and detailed insight into nanoscale phenomena that cannot be captured by traditional macroscopic tests [38]. Techniques used for characterization of corrosion properties of nanomaterial-based systems can broadly be categorized into advanced microscopy techniques and conventional electrochemical techniques. Advanced microscopy includes techniques like Atomic Force Microscopy (AFM) for surface corrosion monitoring, as well as high-resolution liquid cell Transmission Electron Microscopy (TEM) for real-time observation of corrosion pathways at atomic levels [39,40]. Techniques like Scanning Kelvin Probe Force Microscopy and Scanning Electrochemical Microscopy (SECM) are also crucial for studying localized electrochemical behavior and potential differences at the nanoscale [30,41,42]. Conventional electrochemical techniques like Electrochemical Impedance Spectroscopy (EIS) and potentiodynamic polarization are extensively used, providing quantitative data on corrosion rates and mechanisms at different scales, including insights into small-scale electrochemical processes when applied appropriately [23,25,29,41]. Nanoindentation has been found to be invaluable for determining a wide range of properties at the nanoscale, including hardness, modulus of elasticity, creep behavior, strain rate sensitivity, and viscoelastic properties, often employing methods like load–displacement curve analysis or partial unloading tests [25,43,44,45,46,47,48]. Nanoscratch testing is another critical method used to investigate delamination energy, scratch resistance, changes in mechanical properties along the surface, and adhesive strength, providing quantifiable metrics like critical load and allowing analysis of damage shapes and wear mechanisms [29,43,49]. Nano-DMA (Dynamic Mechanical Analysis), often performed with a nanoindenter, applies a sinusoidal stress to characterize viscoelastic properties such as storage modulus, loss modulus, and loss factor (tan δ) as a function of parameters like frequency, temperature, or contact depth, offering insights into viscoelastic behavior and potentially assessing corrosion-induced damage in case of polymer-based matrix [25,29]. Friction Force Microscopy (FFM) or Lateral Force Microscopy (LFM), an advanced mode of Atomic Force Microscopy (AFM), has been an essential tool for high-resolution imaging and characterization of surfaces at nanoscale, enabling visualization of wear features, measurement of wear depth, mapping of frictional and adhesive forces, and analysis of wear tracks and mechanisms, proving indispensable for understanding nanoscale friction, adhesion, and wear phenomena [29,31].

Computational materials science plays a crucial role in investigating nanoscale corrosion, mechanical, and tribological behaviors of nanomaterial-based systems by enabling atomic- to multiscale modeling of material properties and mechanisms. It allows detailed understanding of oxidation and corrosion processes at the atomic level, including surface reactions, defect-promoted oxidation, and size-dependent electrochemical behavior of nanoparticles, which are often distinct from bulk materials [50,51,52]. Additionally, computational studies guide the design of advanced self-healing anti-corrosion coatings by revealing molecular-level healing mechanisms and enabling the prediction of macroscopic coating performance [53]. Molecular dynamics and density functional theory simulations provide insights into mechanical degradation mechanisms such as stress corrosion and fracture toughness reduction in nanostructured materials [54]. Computational approaches also elucidate tribological mechanisms at the nanoscale, including friction reduction by 2D nanomaterials and the influence of surface roughness and indentation depth on wear behavior [55]. Machine learning models integrated with experimental data further enhance the prediction and optimization of corrosion resistance and tribological performance in nanocomposites [56]. However, researchers face challenges in characterizing nanoscale corrosion, including the lack of capability for direct observation with sufficient spatial and temporal resolution to fully unveil mechanisms [39] and the fundamental problem with determining atomic structure at the nanoscale [57]. Characterizing nanomaterials’ mechanical and tribological behavior at the nanoscale also presents several challenges, including the difficulty in standardizing test equipment [29], the limited comparative data available for certain techniques like nanoscratching versus nanoindentation for delamination energy [43], and the inherent complexities of accurate measurement at the nanoscale due to phenomena like the indentation size effect, surface influence, and the presence of residual stresses [38]. Achieving uniform deposition and structure, especially in multilayered nanomaterials like graphene on metal substrates, also poses a significant challenge [45], and the non-uniform distribution or agglomeration of nanoparticles within composites can negatively impact mechanical properties [37,58]. Overcoming these challenges is paramount because studying nanomaterial-based systems’ corrosion, mechanical and tribological properties at the nanoscale holds immense significance, and it is also crucial for optimizing performance, extending lifespan, improving durability, offering significant cost savings, and promoting environmental sustainability.

The primary objective of this review is to bridge the knowledge gap between the unique physicochemical behaviors of nanomaterial-based systems and the advanced metrological tools required to quantify them. This article provides a comprehensive examination of the state-of-the-art in nanomaterial-based systems characterization, organized into three distinct sections following this introduction. Section 2 elucidates the fundamental nanoscale mechanisms governing corrosion resistance, mechanical strengthening, and tribological performance, highlighting specific phenomena such as active corrosion inhibition, confined layer slip, and tribo-film formation. Section 3 subsequently details the review of advanced experimental techniques—ranging from high-speed atomic force microscopy and in situ liquid cell electron microscopy to nanoindentation and nanoscratch testing—to probe nanoscale mechanisms and quantifiable properties. It also addresses the integral role of computational materials science in predictive modeling. Finally, Section 4 critically assesses the persistent challenges in nanoscale experimental characterization, which include substrate effects, instrumentation limits, sample heterogeneity, etc., before concluding with a perspective on future research directions and the necessary standardization of testing protocols.

## 2. Nanoscale Corrosion, Mechanical, and Tribological Mechanisms of Nanomaterial-Based Systems

### 2.1. Corrosion Mechanism of Nanomaterial-Based Systems

The integration of nanomaterials into advanced systems, whether as coatings, films, or bulk nanostructured alloys, represents a paradigm shift in materials science, offering profound enhancements in mechanical and protective properties. This structural refinement, however, fundamentally alters the nature of material failure. The very features engineered for strength—such as high-density grain boundaries, vast nanoparticle–matrix interfacial areas, and precisely architected precipitates—concurrently introduce new electrochemical heterogeneities. These sites become deterministic, rather than stochastic, focal points for unique nanoscale corrosion phenotypes. Pitting corrosion in nanomaterials reflects a localized corrosion phenomenon that results in the development of small pits or cavities on the surface of the material [39,59,60,61]. High-resolution in situ Liquid Cell-Transmission Electron Microscopy (LC-TEM) studies on Sn@Ni_3_Sn_4_ nanocrystals have provided direct, real-time evidence that pitting is deterministically initiated at nanoscale defect sites (voids or cracks) within the protective Ni_3_Sn_4_ layer itself [39]. This finding is critical, as it pinpoints the failure initiation site to the nanoscale heterogeneity within the protective system. The concept of pitting evolves in complex nanostructured alloys. In studies of Al_x_CoCrFeNi HEAs, in situ electrochemical atomic force microscopy (EC-AFM) revealed that as the aluminum content increases, the alloy’s microstructure shifts from a single-phase solid solution to a multiphase system [62]. While the single-phase alloy exhibits conventional pitting, the multiphase alloy does not. Instead, corrosion initiates as a preferential dissolution of the more active Al-rich nanophase or, more commonly, at the nanoscale phase boundaries. In ultrafine grain (UFG) materials such as Al-Zn-Mg alloys, localized corrosion is driven by micro-galvanic events that arise from microstructural inhomogeneities, including grain boundaries and intermetallic particles. The occurrence of these events results in the formation of localized anodic and cathodic sites, which subsequently lead to pitting [39].

Intergranular corrosion (IGC) represents a specific type of localized corrosion that targets the grain boundaries of a metal or alloy, while the interior of the grains remains largely unscathed [63,64,65,66]. In many high-strength alloys, IGC is directly linked to chemical segregation at GBs. In 2024 series aluminum alloys, for example, the susceptibility to IGC is driven by the formation of nanoscale precipitates like S-phase, Al_2_CuMg, along the grain boundaries [67]. These precipitates create a localized micro-galvanic cell with the adjacent precipitate-free zone (PFZ), causing the PFZ to corrode preferentially and the crack to propagate along the grain boundary. The most advanced research shows that not all grain boundaries are equal [68]. A study combining in situ Electrochemical Scanning Tunneling Microscopy (ECSTM) and Electron Back-Scatter Diffraction (EBSD) on nanocrystalline copper revealed that nanoscale IGC initiation is highly dependent on the GB character [68]. High-angle, random grain boundaries were found to be highly susceptible to corrosion initiation. Conversely, specific Coincident Site Lattice (CSL) boundaries were highly resistant. This finding suggests that the corrosion resistance of future nanocrystalline alloys may be improved not by simply reducing grain size but by actively engineering the GB network to maximize low-energy, corrosion-resistant CSL boundaries. Through the application of polarization curves and the assessment of corrosion current densities, Fu et al. [65] demonstrated that nanocomposites containing higher amounts of SiO_2_ are associated with increased corrosion rates. The results indicated an elevated corrosion current density, suggesting that there was a more pronounced active dissolution at the interfaces, especially along the grain boundaries, which points to the incidence of intergranular corrosion.

Galvanic corrosion refers to the accelerated corrosion that takes place when multiple metals with varying corrosion potentials are electrically connected in a corrosive environment. The primary driving force behind this phenomenon is the potential difference between the two metals in a conductive medium. In cases of coupling, the metal exhibits more negative potential functions like the anode, experiencing increased corrosion, whereas the metal with a more positive potential operates as the cathode, where corrosion is mitigated [42,69,70,71]. Coupling Titanium-TC4 with 304 stainless steel in a simulated deep-sea environment illustrates this complex behavior, as the corrosion potential of TC4 may become more positive than that of 304 SS over time, resulting in an anode-to-cathode reversal [69]. The area ratio of the coupled metals significantly determines the designation of anode and cathode, as well as the resultant corrosion behavior, illustrating cases where TC4 or 304 SS consistently assumes either role based on the area proportion. The incorporation of conductive nanofillers like carbon nanotubes (CNTs), graphene, and MXenes into a polymer matrix is a common strategy to enhance barrier properties [72]. However, this creates a significant risk. If these noble, conductive fillers are exposed to the environment, such as via a scratch or poor encapsulation, they form millions of microscopic galvanic cells with the underlying, more active, metal substrate [73]. The nanofiller acts as an efficient local cathode, dramatically accelerating the anodic dissolution (corrosion) of the surrounding metal.

### 2.2. Corrosion Resistance Mechanism Influenced by Nanomaterial-Based Systems

The incorporation of nanomaterials into protective coating systems endows them with advanced functionalities. These functions have evolved far beyond simple passivation, creating multilayered defense strategies. One of the fundamental protection mechanisms involves using nanomaterials to create a superior physical barrier that isolates the metal substrate from the corrosive environment [74]. This is the primary mechanism for nanocomposite coatings, where nanofillers like nanoparticles, nanorods, or nanosheets like nano-clays are dispersed within a polymer matrix [26]. Due to their high aspect ratio and large surface area, these fillers create a complex, maze-like pathway. This “tortuous path” significantly extends the diffusion length and time required for corrosive species like H_2_O, O_2_, and Cl^-^ ions to travel through the coating and reach the metal surface, thereby drastically slowing the corrosion rate [75]. Two-dimensional materials, such as graphene (Gr), hexagonal boron nitride (h-BN), and MoS_2_, represent the theoretical perfect barrier. A single, defect-free layer of graphene is impermeable to all molecules and atoms, including helium [76]. When incorporated into polymer coatings, these 2D nanosheets not only create an exceptionally long tortuous path but also fill and seal the intrinsic nanoscale pores and cracks that exist within the polymer matrix itself, enhancing its barrier properties far beyond what the polymer or fillers could achieve alone [74,76]. Ceramic nanoparticles like silica (SiO_2_)-embedded nanostructured surfaces that mimic the “lotus effect” exhibit water contact angles greater than 150°. This superhydrophobicity provides a physical barrier by trapping a layer of air within the nanoscale surface texture. This air layer, known as the Cassie–Baxter state, prevents the aqueous corrosive electrolyte from making physical contact with the substrate. However, this mechanism is mechanically fragile and can be compromised (leading to a Wenzel state) by physical pressure or even during certain electrochemical measurements [25,77].

Another crucial mechanism is active corrosion protection, where nanomaterials serve as carriers for corrosion inhibitors or possess inherent inhibitory properties themselves, releasing these agents in response to specific stimuli from the corrosive environment [22,78]. These inhibitors absorb onto the metal surface, forming a protective barrier that impedes corrosive processes [22]. Sometimes active protection is achieved via smart nanocontainer-based inhibitor release. In this method, inhibitors are encapsulated within nanoscale carriers like mesoporous silica nanoparticles (MSN) or halloysite nanotubes (HNTs), which are then engineered for stimuli-responsive release [79]. This release can be triggered by mechanical rupture [80] or, more advanced, by a gatekeeper mechanism by polydopamine that responds to a local pH change —the chemical signature of corrosion initiation [81]. When corrosion initiates at a defect, the local anodic activity (metal dissolution) or cathodic activity (oxygen reduction) causes a local change in pH. This pH shift triggers the PDA “gate” to open, releasing the inhibitor only at the active corrosion site [81,82]. Oxide-based carriers, such as cerium oxide (CeO_2_), are known to not only function as containers for corrosion inhibitors but also act as surface modifiers, and CeO_2_ specifically can release cerium ions under acidic conditions, which then react with hydroxyl anions to form protective cerium hydroxides, providing cathodic protection [83,84,85].

Furthermore, nanomaterials enable self-healing capabilities in coatings, allowing them to autonomously repair damage induced by corrosion or mechanical stress, thereby extending the lifespan of the material and reducing maintenance costs [22,28,30]. This often involves nanocontainers embedded in the coating matrix that release encapsulated healing agents or corrosion inhibitors when the coating is damaged or corrosion initiates [28,30,78]. Core–shell nanofibers can be designed to encapsulate both corrosion inhibitors and self-healing agents like linseed oil (LO). When cracks occur, the LO is released by rupturing the nanofibers and can react with oxygen to cure the cracks, while the inhibitor is released in response to environmental cues like pH changes [30]. Another interesting example is Shape Memory Polymers (SMPs). These polymers are programmed with a memory of their original, flat shape [82,86]. When the coating is physically scratched or damaged (a temporary shape), a stimulus such as heat (e.g., T > Tg) provides the molecules with the mobility to overcome internal constraints and return to their original, thermodynamically stable shape, effectively “closing” the crack [82,86]. The frontier of corrosion protection lies in the synergistic interplay of these mechanisms, creating multifunctional systems. A powerful case study is the development of a nanocomposite coating incorporating 8-hydroxyquinoline-5-sulfonic acid-zinc-doped polyaniline (HQZn-PA) into an epoxy matrix [87]. The researchers hypothesized that this complex formulation would create a synergistic system where each component provides a distinct but complementary protective function. The epoxy matrix and the polyaniline (PA) itself form a robust physical barrier. The 8-hydroxyquinoline-5-sulfonic acid (HQZn) component functions as a potent organic corrosion inhibitor, which can be released to passivate the metal surface upon damage. The system possesses two distinct electrochemical healing functions operating in parallel. The conductive polyaniline (PA) provides anodic protection, using its redox activity to hold the metal in a passive state. Simultaneously, the zinc cations provide cathodic protection, acting as a sacrificial anode to protect the exposed steel. This multilayered system (Passive Barrier + Active Inhibitor + Anodic Protection + Cathodic Protection) demonstrated outstanding performance. It reduced the corrosion rate of the epoxy coating by 450-fold and achieved a 99.28% self-healing efficiency in scratched samples. This confirms that the interplay between passive, active, and electrochemical mechanisms is the key to developing next-generation, long-duration protective systems.

### 2.3. Mechanical Strengthening Mechanism of Nanomaterial-Based Systems

The behavior of nanomaterial-based systems at the nanoscale is significantly influenced by factors such as grain size, layer thickness, and the presence of interfaces. Grain boundary strengthening, often referred to as Hall–Petch strengthening, takes place when the grain size of a material is reduced to the nanoscale. The higher density of grain boundaries obstructs dislocation motion, consequently improving the strength of the material. In nanomaterials, grain boundaries serve as obstacles to dislocation motion, which is a fundamental mechanism of plastic deformation [88]. In 0D nanomaterials (nanoparticles), strengthening often results from the dispersion of hard particles at grain boundaries, which block dislocation movement and refine grains, as seen in Ni/SiC nanocomposite coatings, where SiC nanoparticles enhance hardness through Hall–Petch and Orowan mechanisms [89]. For 1D nanomaterials (nanowires or nanotubes), the introduction of features like nanotwins within grains can further inhibit grain boundary migration and dislocation slip, leading to dual strengthening via local dislocation movement and the formation of Lomer–Cottrell locks, as demonstrated in NiCoAl alloys [90]. In 2D nanomaterials (nanosheets, such as graphene), coatings reinforced with graphene or silver-coated graphene nanosheets show grain boundary strengthening by promoting the formation of in situ particles (e.g., TiC) along grain boundaries, which refine grains and increase dislocation density, thereby enhancing strength [91]. For 3D nanomaterials (nanocrystalline or bulk nanostructured materials), grain boundary segregation—such as the introduction of carbon chains or solute atoms like Fe or Zr—can stabilize grain boundaries, pin dislocations, and even induce amorphization at extremely fine grain sizes, all of which significantly boost mechanical strength [92,93,94]. Specific examples include nanocrystalline NiTi, where high-density dislocations and grain boundaries suppress transformation-induced dislocations for stable, high-strength performance [95], and CrCu coatings, where nm-sized Cu precipitates at grain boundaries enhance fracture toughness and hardness [96]. Overall, grain boundary strengthening in nanomaterial coatings is achieved through a combination of grain refinement, particle or solute segregation, and the introduction of structural features that hinder dislocation motion and grain boundary migration.

The confined layered strengthening mechanism in nanomaterial-based systems primarily arises from the restriction of dislocation motion and plastic deformation within ultrathin layers, leading to enhanced strength and toughness. In metallic nanolaminates such as Ni/Al, Ta/Co, and Nb-based systems, as the individual layer thickness decreases to the nanometer scale, dislocations are confined between interfaces, making it difficult for them to propagate freely—this is known as the confined layer slip (CLS) mechanism, which results in significant strengthening as layer thickness is reduced [97,98,99,100]. In Ni/Al nanolaminates, reducing the Al layer thickness from ~110 nm to ~16 nm increases its yield strength by about 68%, following the CLS model [98]. Study on nanolaminate tantalum (Ta)/cobalt (Co) composite (NTCC) [99] revealed evidence supporting the CLS mechanism. The microstructural variations and the properties of the interfaces exert a significant influence. The plastic flow of the NTCCs is influenced by the softer, ductile Co constituent. Consequently, the critical load necessary for dislocation glide in the soft Co layer can be estimated, and this value changes as h varies from 100 nm to 5 nm. The paper indicates that the CLS mechanism functions efficiently at a layer thickness of 10 nm (h). At this scale, the yield strength of the nanolaminate composites aligns with the trend anticipated by the CLS mechanism, underscoring its prevailing influence in this range. The yield strength obtained from the CLS mechanism rises as layer thickness (h) decreases, which is consistent with the observed enhancement in nano-hardness and the associated yield strength of the NTCCs when ‘h’ is lowered from 100 nm to 5 nm [99]. In crystalline/amorphous nanolaminate (e.g., Cu/CuZr), the interfaces mediate plasticity by enabling mechanisms such as shear transformation zone (STZ) activation and dislocation nucleation, allowing plastic strain to traverse interfaces and form shear bands, especially at ultrathin layer thicknesses (~1–2 nm) [97,101]. Graphene–metal nanolayered composites also benefit from confined strengthening, where graphene interfaces block dislocation motion and shield cracks, further enhancing strength and toughness; the efficiency of this mechanism can be tuned by adjusting the Poisson’s ratio of the metal matrix [102]. These examples illustrate that confined layered strengthening in nanomaterials is a universal mechanism, highly dependent on layer thickness, interface structure, and the nature of the constituent phases, enabling the design of materials with superior mechanical properties.

In nanomaterial-based systems, solid solution strengthening is observed when solute atoms are incorporated into a host material, leading to lattice distortions that arise from the discrepancies in atomic size and electronic structure between the solute and host atoms. The distortions generate stress fields that serve as obstacles to dislocation movement, a fundamental mechanism of deformation in crystalline materials [103,104,105]. At the nanoscale, solid solution strengthening plays a crucial role in influencing the nanomechanical properties of materials. In their research on Cu_x_Ni_100−x_/Ta nano-multilayer materials, Shi et al. [103] incorporated Ni atoms into the Cu monolayer of Cu/Ta nano-multilayer materials (NMMs) as a strategy for solid solution strengthening. The study presents microstructural evidence supporting the solid solution strengthening mechanism by analyzing dislocation line lengths. The dislocation line length in CuNi alloy layers tends to be shorter compared to that in pure Cu layers. This indicates that the alloying technique effectively reduces defect formation, which is a crucial element of solid solution strengthening. The incorporation of Ni atoms leads to an enhancement in the hardness of the Cu_x_Ni_100−x_/Ta NMMs. This can be attributed to the greater intrinsic hardness of Ni in comparison to Cu, which effectively illustrates the alloy strengthening mechanism. Li et al. [104] conducted a study on the influence of boron (B) and rare earth elements, specifically neodymium (Nd), on the (CoCrNi)_100−x−y_B_x_Nd_y_ medium-entropy alloy films (MEAFs). The study presents microstructural evidence supporting solid solution strengthening via the refinement of columnar grains. The introduction of trace amounts of boron doping leads to a refinement of the columnar grains in the CoCrNi film, highlighting the underlying strengthening mechanism. Neodymium (Nd) plays a significant role in enhancing solid solution strengthening. The discrepancy in size between Nd and the host lattice atoms leads to lattice distortions that hinder dislocation motion. This effect demonstrates efficacy even at lower concentrations of Nd, approximately 1–3%. Boron atoms, compared to neodymium, function as interstitial atoms because of their smaller atomic radius. The presence of these atoms occupies the interstitial spaces among the larger metal atoms in the alloy, effectively impeding dislocation movement and thereby improving the mechanical properties of the material. The highest hardness recorded was 13.28 GPa in MEAFs that had slight B doping and no Nd. Study led by Xu et al. [105] provides a significant contribution to the understanding of solid solution strengthening in Mg-Bi and Mg-Al-Bi alloys by employing a high-throughput experimental approach, where a diffusion couple was created to have a continuous gradient of Bi and Al concentrations. By creating a continuous concentration gradient of Bi and Al within single samples and then conducting a series of nanoindentation tests along this gradient, they efficiently mapped the mechanical response to alloying. The results confirmed that Bi has a slightly greater strengthening effect on hardness than Al, which aligns with theoretical predictions based on atomic size differences and electron work function (EWF) values. This work effectively confirmed the solid solution strengthening mechanism by showing a clear, quantifiable cause-and-effect relationship: adding more solute atoms to the magnesium solid solution makes the material harder.

In practice, the exceptional mechanical properties of any advanced NBS arise not from a single mechanism but from the synergistic interplay of multiple, simultaneously acting mechanisms. A 2D graphene–Ni nanocomposite is strengthened by (1) confined layer slip (CLS) as dislocations are blocked by the 2D graphene planes, (2) Grain Boundary Strengthening, because the graphene sheets also act as Zener pinning agents to refine the matrix grain size, and (3) enhanced work hardening, as the CLS mechanism itself rapidly generates a high density of trapped dislocations [106]. Even simple 0D TiC-nanoparticle-reinforced Ni coating exhibits a sophisticated synergy: (1) Orowan Looping occurs as dislocations bow around the TiC particles, while (2) Grain Boundary Strengthening is active in the nanocrystalline Ni matrix. The crucial interplay is that the 0D TiC particles enable the GBS by pinning the Ni grains at high temperatures, preventing grain growth and preserving the fine-grained structure [107]. Understanding this interplay is the central challenge and opportunity in designing next-generation, high-strength nanomaterial systems.

### 2.4. Tribological Wear Mechanism of Nanomaterial-Based Systems

Abrasive wear in nanomaterial-based systems typically occurs when hard particles or asperities slide against a surface, causing material removal through micro-cutting, plowing, or scratching. In metal matrix nanocomposites (MMNCs), abrasion is often the dominant wear mechanism, especially when reinforced with ceramic nanoparticles, which enhance wear resistance by increasing hardness and impeding the movement of abrasive particles across the surface [108]. In AZ31 magnesium alloy nanocomposites reinforced with tungsten carbide (WC) and graphite nanoparticles, abrasion and oxidation were identified as the main wear mechanisms, with the addition of just 1 wt.% graphite nanoparticles significantly improving wear resistance by reducing the extent of abrasive damage [109]. In polymer nanocomposites, such as low-density polyethylene (LDPE), nanoscale abrasive wear can manifest as plowing (material displaced to the sides) or cutting (material removed from the surface), with environmental factors like UV weathering shifting the dominant mechanism from plowing to cutting, thereby increasing wear rates [110]. The inclusion of nanomaterials like carbon nanotubes in lubricants or as reinforcements can also provide a shielding effect, reducing the cutting action of abrasive particles and lowering overall wear by up to 35% [111]. The unique atomic-thin, layered structure of 2D materials like graphene, MoS_2_, and hexagonal boron nitride allows them to act as solid lubricants, reducing direct contact and thus minimizing abrasive wear by forming a protective tribo-film or transfer film on the surface [17,112,113,114]. For example, when MoS_2_/reduced graphene oxide (RGO) nanohybrids are incorporated into a phenolic resin composite, the wear mechanism shifts from severe abrasive and adhesive wear (in pure resin) to much milder abrasive wear, as the 2D nanomaterials distribute load, repair surface defects, and form a stable transfer film that protects the underlying material [115]. Surface treatments with graphene and h-BN on steel have been shown to decrease both the friction coefficient and wear area significantly, with the wear mechanism attributed to the formation of a tribo-film that resists abrasive action [112]. In polymer nanocomposites, 2D carbon nanostructures like graphene can reduce abrasive wear rates by over 70% compared to unreinforced polymers, mainly due to their ability to prevent direct hard contact and dissipate stress [116]. The tribological performance of MoS_2_ is fundamentally governed by its ability to form a low-shear, lamellar tribo-film at the sliding interface. One study demonstrates that MoS_2_ nanosheet morphology is superior to nanoflowers as an additive, as the nanosheets more effectively exfoliate to create a continuous film and reduce wear [117]. This lubricating film, however, is highly susceptible to environmental degradation from humidity. Water molecules are shown to preferentially adsorb onto S-vacancy defects and, crucially, onto lamellae edges. This edge adsorption is detrimental, as it negatively impacts the tribological performance by inhibiting the formation of an ordered, low-friction tribo-layer [118]. Oxygen bonding with MoS_2_ plays a complex role in their tribological (friction and wear) performance. Traditionally, oxygen contamination was thought to degrade MoS_2_’s lubricating properties, but recent research shows that oxygen incorporation can lower both friction and wear under certain conditions. Specifically, oxygen can cause amorphization of MoS_2_ coatings, but during sliding, an ultrathin crystalline MoS_2_ tribo-layer with incorporated oxygen forms, which reduces the coefficient of friction and increases wear resistance due to higher film density and hardness [119]. However, the effect of oxygen depends on the structure and environment: amorphous MoS_2_ is more susceptible to oxidation and wear, while highly ordered crystalline films or those with higher density resist oxygen diffusion and maintain better tribological properties [120,121].

The adherent wear mechanism in nanomaterial-based systems involves the transfer and adhesion of material from one surface to another during sliding contact, often leading to the formation of a transfer layer or tribo-layer that can influence friction and wear behavior. In nanocomposite films such as NbN and NbN/NbN-Ag, adhesive wear is a dominant mechanism, where material from the film adheres to the counterface (e.g., Al_2_O_3_ ball), resulting in material transfer and the formation of a protective layer that can reduce further wear, especially when combined with oxidation processes [122]. For instance, in NbN/NbN-Ag multilayer nanocomposite films, adhesive and oxidation wear were observed as the main mechanisms, with the formation of a tribo-layer that helped improve wear resistance compared to films with only Ag doping. Oxygen bonding with nitride-based nanomaterials, such as during plasma oxy-nitriding, leads to the formation of surface layers containing oxynitride phases like iron or chromium oxy-nitrides and nanocrystalline structures that significantly influence wear resistance. Plasma oxy-nitride steel develops an iron oxide layer with nanocrystalline Fe_2–3_N phases, and the optimal addition of oxygen during treatment results in a thicker, harder surface layer that improves both wear and corrosion resistance compared to conventional nitriding [123]. Similarly, oxy-nitriding AISI 304 stainless steels via plasma electrolytic methods produces a surface with small craters of chromium and iron oxy-nitrides and a subsurface nitrogen diffusion layer, which together can triple the wear resistance relative to untreated steel [124]. In MXene-based coatings (e.g., Ti_3_C_2_T_x_), the wear process leads to the formation of a tribo-layer composed of degraded MXenes and iron oxides. This tribo-layer adheres to both the coating and the counterbody, transforming the contact interface and enabling low shear resistance and ultra-wear-resistant performance. The continuous supply of fresh nanosheets from MXene pileups at the wear track further supports the adherent wear mechanism by maintaining a lubricious interface [125]. In polymer-derived nano-ceramic composites, the wear mechanism shifts from abrasive to adhesive under specific conditions, particularly when severe plastic deformation occurs during processing, thereby improving the bonding between the ceramic and metal matrix [126]. In natural rubber nanocomposites, a notable transition from abrasive to adhesive wear was observed under severe conditions, where a viscous film formed by degradation products significantly influences the wear behavior [127]. In case of composites containing 2D-laminated molybdenum disulfide (MoS_2_) and reduced graphene oxide (RGO), these nanomaterials disperse well within the matrix and, under friction, form a stable and continuous transfer film on the wear surface. This film acts as a protective barrier, bearing more load, repairing surface defects, and reducing direct contact between sliding surfaces, which leads to lower friction and wear rates compared to systems without such nanomaterials [113,115,128]. The addition of just 0.10 wt.% of the nanohybrid significantly decreased the friction coefficient and wear rate, shifting the wear mode from severe fatigue and adhesive wear to milder abrasive and adhesive wear. The synergistic effect of the 2D structure and strong interfacial bonding enables the nanomaterials to form a robust adherent layer, which is key to their anti-wear performance [115].

Atomic attrition corresponds to a wear mechanism that manifests at the nanoscale, defined by the gradual removal of material on an atomic level. This process differs from macroscopic wear mechanisms, which typically entail larger-scale material removal via processes such as fracture or plastic deformation. At the atomic scale, the mechanisms of wear are influenced by the formation and breaking of atomic bonds at the interface between two materials. This process is frequently affected by stress and the chemical conditions present at the contact interface [129,130,131,132]. Jiang et al. [129] demonstrates that polymer wear is a combination of atomic attrition and viscoelastic relaxation by showing that the standard model for atomic attrition fails to predict wear in polymethylmethacrylate (PMMA) at elevated temperatures. The researchers then successfully account for this discrepancy by modifying the model to include a term for viscoelastic relaxation, which is a temperature-dependent property of polymers. Another intriguing example emerges from the investigation of wear on polycrystalline silicon (poly-Si) surfaces in contact with a SiO_2_ microsphere, emulating the conditions present in chemical mechanical polishing/planarization (CMP) processes [130]. Here, material removal is primarily influenced by stress-assisted mechanochemical reactions that demonstrate atomic attrition behavior. The material removal rate for poly-Si was found to be lower than that of monocrystalline silicon (mono-Si) due to the presence of oxides clustered within the intercrystallite boundaries of poly-Si, which minimizes its chemical reactivity and elevates the energy barrier for mechanochemical reactions.

Beyond the classic mechanisms, two other wear modes are critical to the performance of NBS: tribo-chemical wear and delamination wear. The tribo-corrosion wear mechanism in nanomaterial-based systems like high-entropy alloys and CrN coatings is driven by a powerful synergistic effect where mechanical wear and electrochemical corrosion mutually accelerate each other [133,134,135]. The total material loss is significantly greater than the sum of the individual wear and corrosion processes. The mechanism proceeds as follows: First, at the nanoscale, corrosion initiates at inherent weak points in the material’s protective passive film, such as grain boundaries or phase interfaces. Then, the mechanical action of friction slides over this surface, damaging and removing that thin protective layer. This exposes the fresh, chemically active material underneath directly to the corrosive environment, allowing degradation to occur much more rapidly. This mutual action cycle—where wear removes the protective film and corrosion attacks the exposed material repeatedly—leads to accelerated failure. Delamination wear, also known as fatigue wear, is a mechanism where repeated stress or cyclic loading causes the separation of layers (delamination) within a material, leading to material loss and reduced mechanical integrity. In nanomaterial-based systems, such as metal matrix nanocomposites (MMNCs) and polymer nanocomposites, delamination is a significant concern due to the layered or composite structure. In MMNCs, delamination wear is frequently observed alongside abrasion and adhesion, especially under harsh conditions, and is influenced by factors like reinforcement type, processing method, and nanoparticle size [108]. In polymer nanocomposites, drilling-induced delamination can reduce residual strength, but the incorporation of nanofillers like graphene and montmorillonite clay has been shown to minimize delamination and improve fracture toughness [136]. In metal nanocomposites, delamination is also the dominant wear mechanism at elevated temperatures, as observed in aluminum alloys reinforced with nano-Si_3_N_4_ composite, where delamination wear was prevalent between 50 °C and 250 °C [137].

## 3. Experimental and Computational Methods for Probing Nanoscale Corrosion, Mechanical, and Tribological Mechanisms

### 3.1. Corrosion Measurement Techniques

#### 3.1.1. Advanced Force/Electron Microscopy Techniques

**Figure 1:**
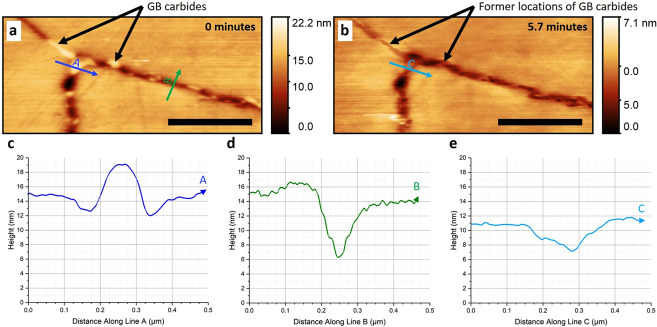
Images show HS-AFM topographic maps from a time-lapse of thermally sensitized AISI Type 304 stainless steel undergoing corrosion within 395 mg L^−1^ aqueous sodium thiosulfate at: (**a**) 0 min, and (**b**) 5.7 min. Also shown are line profiles of height changes collected across: (**c**) Line A indicated in (**a**) as a dark blue line, (**d**) Line B indicated in (**a**) as a green line, (**e**) Line C indicated in (**b**) as a light blue line (Reprinted from ref. [139], Copyright (2024) by the authors, Licensee Springer Nature Limited. This article is an open access article distributed under the terms and conditions of the Creative Commons Attribution (CC BY) license https://creativecommons.org/licenses/by/4.0/ (accessed on 29 November 2025)).

Atomic Force Microscopy (AFM) has become an essential technique for assessing corrosion in nanomaterials, providing exceptional spatial and temporal resolution that is vital for comprehending localized corrosion phenomena. This technique effectively captures the initiation and progression of corrosion at the nanoscale, which is crucial for formulating strategies to prevent material degradation. AFM-based techniques offer comprehensive insights into the surface topography and mechanical properties of materials, allowing researchers to monitor corrosion processes in real-time and across different environmental conditions [138,139,140,141,142,143,144,145]. HS-AFM represents a significant advancement in imaging techniques, offering a speed that exceeds the performance of conventional AFM methods. This technique utilizes a non-resonant probe, allowing for real-time adjustments of the distance between the probe and the sample. This capability facilitates ultrafast scanning and imaging, achieving scanning rates that exceed a thousand pixels per second [138,139,140]. HS-AFM offers nanoscale spatial resolution, crucial for the real-time detection and characterization of localized corrosion sites, including pits and intergranular attacks [140]. Figure 1 presents a time-lapse series of in situ High-Speed Atomic Force Microscopy (HS-AFM) images. These images demonstrate the effect of a corrosive environment (395 mg/L aqueous sodium thiosulfate) on thermally sensitized AISI Type 304 stainless steel sample in the absence of applied stress [139]. Primarily, it was discovered that even without applied stress, the thiosulfate solution causes localized corrosion by preferentially dissolving the carbide precipitates located at the grain boundaries. This process creates micro-pits on the surface. These observations are significant because they identify a potential initiation mechanism for Stress Corrosion Cracking (SCC); these micro-pits could act as stress concentrators when a tensile load is eventually applied to the material.

**Figure 2:**
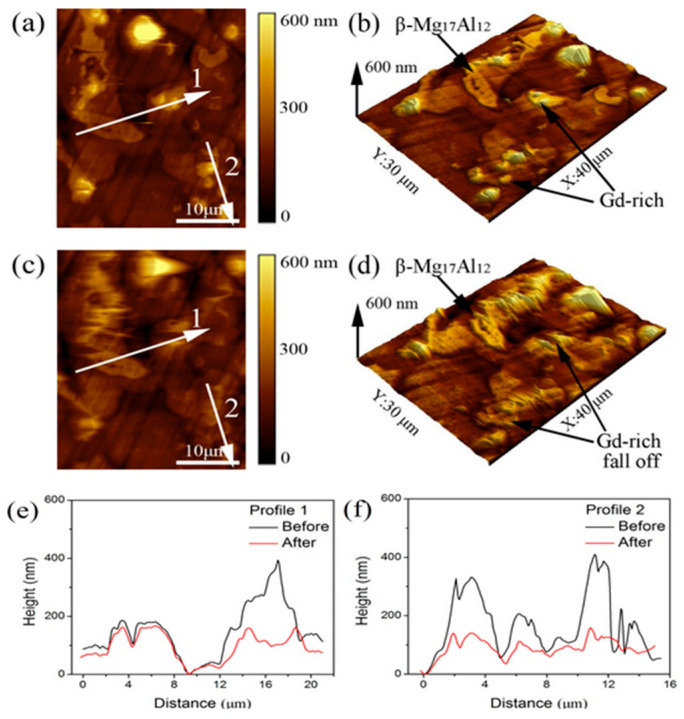
In situ AFM maps of the Mg-9Al-1Fe-1Gd alloy showing corrosion events occurring on adjacent precipitate sites: (**a**,**b**) before corrosion; (**c**,**d**) after 30 min of exposure to a 3.5% wt.% NaCl solution at 25 °C; (**e**,**f**) height profiles for lines 1 and 2 in (**a**,**c**) showing the topographical changes (Reprinted from ref. [142], Copyright (2024) by Chongqing University, Publishing services by Elsevier B.V. on behalf of KeAi Communications Co. Ltd. This article is an open-access article distributed under the terms and conditions of the Creative Commons Attribution (CC BY-NC-ND) license https://creativecommons.org/licenses/by-nc-nd/4.0/ (accessed on 29 November 2025)).

At present, electrochemical atomic force microscopy (EC-AFM) has gained significant traction in the materials science domain. EC-AFM facilitates the initiation of electrochemical reactions through the application of an external potential to the scanning probe. This capability enables AFM to visualize electrochemically active regions on the surface and gather scanning images, which are essential for investigating local chemical reaction behaviors and polarization phenomena [141,142,143,144,145]. In situ EC-AFM corrosion observation of Mg-9Al-1Fe-(Gd) alloys in 3.5 wt.% NaCl solution was conducted, alongside the measurement of the Volta potential of various constituent phases using scanning Kelvin probe force microscopy [142]. The localized micro-galvanic corrosion of Mg-9Al-1Fe-(Gd) alloys in a 3.5 wt.% NaCl solution was investigated using in situ EC-AFM and SKPFM techniques. The Volta potential difference for the AlFe_3_ and β-Mg_17_Al_12_ phases was approximately 400 mV and 150 mV, respectively, in comparison to the α-Mg phase, which measured around 50 mV. This suggests that AlFe_3_/α-Mg and β-Mg_17_Al_12_/α-Mg established two distinct types of micro-galvanic couples. Figure 2 illustrates the in situ EC-AFM corrosion morphology of the Mg-9Al-1Fe-1Gd alloy immersed in a 3.5 wt.% NaCl solution following a duration of 30 min. The arrows in Figure 2a,b indicate the presence of Gd-rich (gadolinium) intermetallic with a diameter of approximately 2 µm, alongside rod-shaped β-Mg_17_Al_12_ phases in the Mg-9Al-1Fe-1Gd alloy. Following a 30 min corrosion of the sample, there was no significant alteration in the profile of the β-Mg_17_Al_12_ phase, as illustrated in Figure 2c,d. The corrosion observed around the β-Mg_17_Al_12_ phase was found to be less severe compared to that surrounding the Gd-rich phase. In the meantime, the anodic α-Mg matrix next to the Gd-rich phase underwent dissolution throughout the corrosion process. Additionally, significant localized corrosion was observed around certain Gd-rich phases in the Mg-9Al-1Fe-1Gd alloy.

Liquid cell transmission electron microscopy (LC-TEM) represents an advanced technique that facilitates the in situ observation of dynamic processes occurring in liquid environments, achieving high spatial and temporal resolutions. This approach proves to be especially useful for investigating phenomena in liquid samples, including crystallization, nanoparticle dynamics, and chemical reactions, which are typically difficult to observe with conventional microscopy methods [146,147,148,149]. This technique has been utilized to investigate the behavior of metal nanoparticles when subjected to high-energy electron irradiation. Observations include morphological changes and transitions between amorphous and crystalline states, underscoring the significance of charge-induced transformations in the evolution of nanoparticles [147]. LC-TEM facilitates the investigation of chemistry and growth kinetics at nanoscale as they relate to temperature variations. Studies on the growth of Ag nanocrystals have revealed notable variations in morphology and growth rates as a function of temperature, contributing to the formulation of kinetic models for reactions that depend on temperature [148]. Electrochemical Scanning Transmission Electron Microscopy (EC-STEM) represents a sophisticated microscopy technique that enables the observation of dynamic processes, including corrosion, as well as the real-time monitoring of alterations in morphology, composition, and crystallography of nanoparticles in situ [150,151,152]. The methodology diverges from LC-TEM, employing a 3-electrode cell system to apply potential to the sample for investigating the electrochemical properties of nanomaterials, whereas LC-TEM examines a broad spectrum of dynamic processes in liquid environments, including nanoparticle synthesis, diffusion, and aggregation.

#### 3.1.2. Conventional Electrochemical Techniques

The investigation of corrosion phenomena has long been the domain of conventional electrochemical techniques. These methods provide the critical, quantitative framework for assessing material degradation. Open circuit potential (OCP) testing is a fundamental electrochemical technique used to assess the surface reactivity, stability, and charge transfer properties of nanomaterial-based systems. This measure establishes the free corrosion potential of a material in a specific electrolyte. It represents a thermodynamic mixed potential, indicating the overall tendency of the material to corrode, but provides no kinetic information [153,154]. OCP testing is widely used to evaluate corrosion behavior, surface passivation, and galvanic coupling in nanomaterial composites, such as Mg-Fe systems and metallic biomaterials. High-resolution OCP scans can reveal local electrochemical activity, phase distribution, and the effects of microstructure on reactivity [155]. Potentiodynamic Polarization (PDP) technique applies a potential sweep to the sample, forcing it away from its equilibrium state to measure the resulting current response. By extrapolating the linear (Tafel) regions of the polarization curve, it provides the fundamental kinetic parameters of corrosion, including the corrosion current density, which is directly proportional to the corrosion rate, as well as the pitting potential and passivation potential [156]. In a study of a new composite 3-methylpyrrole-dodecyl sulfate sodium/3,4-ethylenedioxythiophene (P3MPY-SDS/EDOT) polymer coating on carbon steel, researchers hypothesized the coating would provide effective anti-corrosion protection in a strong acid (0.5 M H_2_SO_4_) [157]. PDP testing confirmed this hypothesis. The results showed that the corrosion rate of the coated sample was nine times lower than the uncoated sample, and an inhibition efficiency of over 90% was achieved. Electroplating of nanocrystalline Ni–Fe monolayer alloy coatings was conducted within a current density range of 2.0 to 5.0 A dm^−2^ [23]. The coatings’ stability was assessed using the potentiodynamic polarization method. The corrosion rates were assessed using the Tafel extrapolation method, alongside measurements of other corrosion parameters such as corrosion potential (E_corr_) and corrosion current density (i_corr_). PDP results clearly illustrated that, among the Ni–Fe alloy coatings deposited at various current densities, the (Ni–Fe)_2.0_ Adm^−2^ alloy coating exhibits the lowest corrosion potential, attributed to its smallest crystallite size (10.4 nm) and the highest Fe content compared to the other deposited coatings.

Electrochemical Impedance Spectroscopy (EIS) is a non-destructive technique. EIS applies a small-amplitude sinusoidal AC potential perturbation over a wide range of frequencies. The resulting impedance data is analyzed using equivalent electrical circuit (EEC) models to extract detailed information about the corrosion mechanism and surface properties. It is the gold standard for evaluating coating integrity, capable of distinguishing the resistance of ion pathways, the dielectric property of the barrier (coating capacitance), and the resistance of the electrochemical reaction at the metal–electrolyte interface (charge transfer resistance) [25,77]. The equivalent circuit models help to quantify parameters such as pore resistance, coating capacitance, and charge transfer resistance, which are directly linked to the protective performance of the nanomaterial system. Enhanced corrosion resistance is typically reflected by increased polarization resistance and total impedance, as well as the appearance of larger semicircles in Nyquist plots, which are attributed to improved barrier effects and self-healing properties of the nanomaterial coatings [158,159,160]. To investigate self-healing capability, EIS was applied to the epoxy coatings with an artificial scratch to simulate damage. The self-healing potential was directly monitored by tracking the change in the coating’s low-frequency impedance modulus over immersion time in the corrosive environment. A significant increase in this impedance value indicated the successful restoration of the barrier properties as the corrosion inhibitors (phosphate and glutamate) were released from the smart carriers into the scratch zone, forming a protective film that sealed the defect [161]. EIS was the primary conventional method used to quantify the overall anti-corrosion property of the composite-doped waterborne epoxy (WEP) coating. The EIS results revealed an extraordinarily high impedance modulus of 9.84 × 10^9^ Ω·cm^−2^ [162]. This value showed highest orders of magnitude than typical WEP coatings and signifies a near-perfect barrier. Salt spray tests confirmed this, with the coating exceeding 30 days of protection. EIS was used to test the hypothesis that the substrate material (carbon steel vs. brass vs. Al alloy) influences the failure of the same epoxy coating [163]. By tracking the EIS impedance modulus at lowest frequency over time, they proved the coating on steel failed fastest, while the coating on the Al alloy lasted the longest. The analysis concluded that the steel’s corrosion products (loose and hygroscopic) actively accelerated the diffusion of water through the coating, whereas the passive film on the Al alloy inhibited this degradation.

### 3.2. Mechanical and Tribological Properties Measurement Techniques

#### 3.2.1. In Situ SEM/TEM Tensile Testing

**Figure 3:**
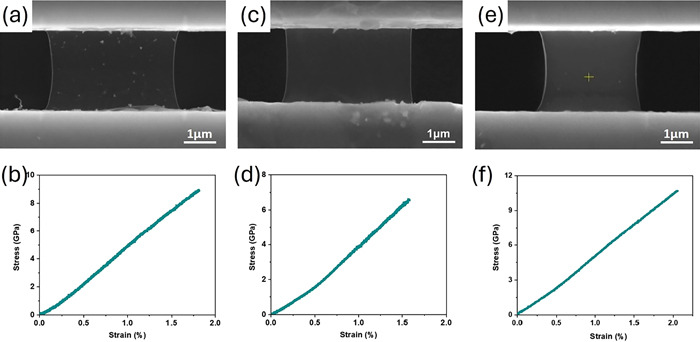
SEM images and corresponding engineering stress–strain curves from tensile-tested h-BN samples with different thicknesses. (**a**,**b**) three layers; (**c**,**d**) five layers; (**e**,**f**) six layers (Reprinted from ref. [172], Copyright (2024) by the authors, Licensee AIP Publishing. This article is an open access article distributed under the terms and conditions of the Creative Commons Attribution (CC BY) license https://creativecommons.org/licenses/by/4.0/ (accessed on 29 November 2025)).

In situ TEM and SEM tensile testing methods are employed to assess the mechanical properties of materials at the micro- and nanoscale by monitoring the deformation process in real time within a transmission electron microscope (TEM) or scanning electron microscope (SEM) [164,165]. In situ SEM tensile tests provide a detailed observation of the sample’s surface morphology and the deformation process involved. In contrast, in situ TEM tensile tests provide valuable insights into the microstructural and phase changes occurring at the atomic level [165,166]. The tests provide comprehensive insights into mechanical properties, such as yield strength, ultimate tensile strength, fracture strength, fatigue resistance, and Young’s modulus. This method additionally offers valuable insights into the mechanisms of deformation and the alterations in microstructure. Results are derived through the analysis of stress–strain curves, fracture morphology, and the microstructural changes noted during the tests [165,167]. In situ SEM and TEM studies of high-entropy alloys and nanolaminates have shown that mechanisms like nanoscale twinning, detwinning, and phase transformation (e.g., FCC to BCC) contribute to enhanced strength and ductility by refining grains and increasing barriers to dislocation movement [168,169,170]. In situ SEM-EBSD and SEM-DIC (Digital Image Correlation) were used to map strain fields and microstructural evolution in dual-phase steels, where martensite strain localizations and ferrite plasticity can be directly correlated with microstructural features at the nanoscale [171]. TEM tensile testing has shown that specific interfaces, such as SiC/MgAl_2_O_4_, demonstrate greater bonding strength relative to others, like MgAl_2_O_4_/Al, attributed to lower degrees of mismatch [166]. This approach is utilized to investigate materials such as silicon nitride whiskers and hexagonal boron nitride (h-BN) nanosheets, yielding insights into their tensile properties and fracture behavior [165,172]. In situ SEM tensile testing has demonstrated that materials such as Si_3_N_4_ whiskers display a linear stress–strain relationship until fracture, suggesting a predominance of elastic deformation. This technique facilitates the examination of microstructural alterations occurring during deformation, including the recovery of lath structures and the coarsening of precipitates in alloys, which may result in mechanical softening [164]. A tensile test using in situ SEM was performed on single-crystal few-layer hexagonal boron nitride (h-BN) nanosheets, which varied in layer numbers from 3 to 8 [172]. Figure 3 presents SEM images alongside the corresponding engineering stress–strain curves derived from tensile tests conducted on h-BN samples of varying thicknesses. The stress–strain curves for all three samples demonstrate a nearly linear relationship, indicating elastic behavior up to the point of fracture.

#### 3.2.2. Hardness and Modulus Properties Measurements

Nanoindentation and AFM-based indentation are both techniques used to measure the mechanical properties of materials at micro- to nanoscale. The operating principle of nanoindentation involves pressing a sharp indenter (often made of diamond) into the material’s surface while continuously recording the applied force and the resulting indentation depth; this data is then analyzed, often using models like Hertzian mechanics, to extract properties such as hardness and Young’s modulus [173,174]. AFM-based indentation uses the tip of an atomic force microscope as the indenter, allowing for extremely high spatial resolution and the ability to probe very small or heterogeneous regions, such as individual crystalline structures or soft biological samples, by measuring the cantilever deflection as the tip interacts with the sample surface [174,175,176,177]. The Oliver–Pharr method is widely employed in both types of nanoindentation techniques for calculating the reduced modulus of elasticity through the analysis of the contact area between the indenter and the material surface [178]. The main outcomes from both methods are quantitative measurements of mechanical properties, including elastic modulus, hardness, and sometimes rupture strength or adhesion energy [177,179,180]. AFM-based indentation offers the added advantage of mapping mechanical heterogeneities at the nanoscale and is particularly useful for thin films, soft materials, or complex composites where high spatial resolution is required [174,175,176,179]. Nanoindentation is generally more suitable for bulk or larger-scale measurements, but both methods can be complementary; for example, combining them can provide a more complete mechanical characterization of complex materials like shale or polymer composites [173,174,179]. Nanoindentation hardness and modulus measurements are essential for understanding and quantifying the mechanical strengthening mechanisms in nanomaterial-based systems. These measurements provide localized data on how different phases, grain sizes, or compositional changes affect properties like hardness and elastic modulus, which are directly linked to strengthening mechanisms such as solid solution strengthening, grain boundary strengthening, and precipitation hardening. In CoCrNi medium-entropy alloys, nanoindentation revealed that increasing chemical short-range order (SRO) leads to higher hardness and strength due to enhanced dislocation pinning and interaction, with hardness increasing by up to 13.7% compared to random solid solutions [181]. Similarly, in CoCrNiMox high-entropy alloys, nanoindentation mapping showed that the addition of Mo increases hardness in the FCC phase via solute strengthening, while the formation of a Mo-rich Laves phase results in even higher hardness, illustrating how phase composition and distribution contribute to overall strengthening [182]. In nanocomposites, such as graphene nanoplatelet-reinforced nickel aluminum bronze, nanoindentation demonstrated that increasing nanoparticle content improves nano-hardness and modulus, indicating enhanced resistance to deformation and improved mechanical performance due to effective load transfer and matrix reinforcement [183]. High-throughput nanoindentation mapping in additively manufactured Ni superalloys has also been used to resolve microscale strength variations and identify the effects of precipitation and chemical segregation on local hardness, directly linking these features to specific strengthening mechanisms like precipitation hardening and coherency strengthening [184]. In chromium carbide–nickel chromium (Cr_3_C_2_-NiCr) coatings, the hardness maps show a strong correlation with microstructural variations, offering valuable insights into phase distribution and the evolution of mechanical properties [185]. This technique proves to be highly effective in bridging the gap between the heterogeneous microstructure and the localized mechanical performance at the nanoscale.

#### 3.2.3. Viscoelastic Properties Measurements

**Figure 4:**
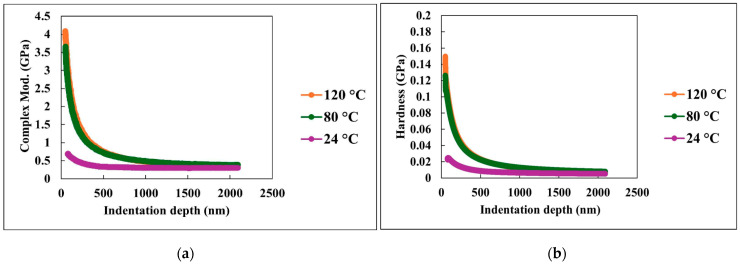
(**a**) Complex modulus versus indentation depth at various temperatures, (**b**) hardness versus indentation depth at various temperatures (Reprinted from ref. [194], copyright (2025) by the authors and Licensee MDPI, Basel, Switzerland. This article is an open access article distributed under the terms and conditions of the Creative Commons Attribution (CC BY) license https://creativecommons.org/licenses/by/4.0/ (accessed on 29 November 2025)).

Many advanced nanomaterial-based systems exhibit viscoelastic behavior—a time-dependent response that combines the characteristics of an elastic solid and a viscous fluid. Understanding this behavior is critical, as it governs energy dissipation, damping, and impact resistance [186,187,188]. Viscoelastic properties are measured using dynamic mechanical analysis, which, at the nanoscale, is performed using dynamic nanoindentation (CSM) or dynamic AFM modes like contact resonance AFM. These methods allow for the quantification of viscoelastic parameters like storage modulus, loss modulus, and loss tangent, even in very small volumes or thin films, and are supported by both experimental and theoretical modeling approaches [187,189]. Measuring viscoelastic properties reveals mechanical strengthening mechanisms in nanomaterial-based systems by linking energy storage, dissipation, and interfacial effects to material structure. Adding graphene nanoplatelets (GNP) to epoxy increases the storage modulus, indicating improved stiffness due to better load transfer and matrix reinforcement. The loss modulus and tan δ also change, which reflects enhanced energy dissipation and interfacial effects, which are crucial for toughness and damping [190,191]. In hybrid systems with montmorillonite (MMT) nanoclay and multi-walled carbon nanotubes (MWCNT), the storage modulus increases due to restricted polymer chain mobility, while the loss modulus peak broadens, indicating more effective energy dissipation and stress transfer at the filler-matrix interface [192]. In the case of polymer nanocomposites, it is observed that the storage modulus rises with frequency, whereas the loss modulus declines, suggesting a transition from viscous to elastic behavior [193]. The results showed that the incorporation of alumina nanoparticles (NPs) leads to a substantial increase in both the storage modulus (E′), representing stiffness, and the loss modulus (E″), representing energy dissipation. This stiffening effect was more pronounced at higher frequencies and was most efficiently achieved with the smaller 25 nm particles. However, a crucial finding was that the overall damping capacity (tan δ) consistently decreased with the addition of NPs. This is attributed to the fact that the increase in stiffness far outweighed the increase in energy dissipation, causing the composite to behave in a more elastic and less viscous manner. Yao et al. [194] investigated nanoscale viscoelastic properties of silica nanoparticle-reinforced superhydrophobic coating to investigate the effect of temperature. Nano-DMA results shown in Figure 4 demonstrate that the nanoparticle-reinforced coating’s properties are highly dependent on both indentation depth and temperature. Both the complex modulus and hardness were highest at the surface and decreased as the indenter penetrated deeper, indicating a more reinforced surface layer compared to the bulk material. This effect was strongly influenced by temperature; as the testing temperature was increased from 24 °C to 120 °C, both the hardness and complex modulus values decreased substantially at all depths. The thermally induced softening of the polymer matrix was the primary cause for this decline in mechanical performance.

#### 3.2.4. Time-Dependent Nanomechanical Properties Measurements

Creep, stress relaxation, and strain rate sensitivity tests are crucial for comprehending the time-dependent mechanical behavior of nanomaterial-based systems at the nanoscale. These tests offer valuable insights into the deformation behavior of nanomaterials under sustained stress, their stress relaxation characteristics over time, and their sensitivity to variations in strain rate, respectively. Nanoindentation serves as a prevalent technique for performing creep tests at the nanoscale. The test is conducted in load-control mode, maintaining a constant load while recording the displacement to examine the creep behavior [44]. This holds significant relevance for materials such as supercrystalline nanocomposites (SCNCs) and shale, where the phenomenon of creep can be affected by periodic microstructural characteristics. Nanoindentation creep tests serve to assess the mechanical properties of materials such as shale and polymer nanocomposites, offering valuable insights into their long-term stability [47,195]. In Ni-based superalloys and concentrated solid solution alloys, nanoindentation creep tests identify dislocation-controlled mechanisms as dominant, with alloying elements like Cr, Mn, and Fe providing significant strengthening by increasing lattice distortion and impeding dislocation motion [196,197]. For nanocomposites such as sintered silver doped with carbon nanotubes and PDMS–silica coatings, nanoindentation creep tests reveal how nanofillers and temperature affect hardness, creep rate, and energy dissipation, providing insights for optimizing mechanical performance [198,199]. The stress relaxation test necessitates the application of constant strain to a material, followed by an observation of the stress drop that occurs over time. Materials that contain a larger number of mobile dislocations and impurities demonstrate increased stress relaxation, since these dislocations can move more freely when subjected to stress [46,200]. The response of a material to stress relaxation can be effectively quantified by creating a plot of logarithmic stress versus logarithmic strain rate. Stress relaxation tests also allow visualization and analysis of internal defect evolution and stress distribution, as demonstrated in polycrystalline γ-TiAl alloys, where the load stabilizes after an initial drop, providing insight into the mechanisms of load change and defect dynamics during relaxation [201]. The strain rate sensitivity test evaluates the variation in flow stress of a material as strain rates change. The test is generally performed through nanoindentation techniques, in which the strain rate is adjusted, and the resulting variations in hardness or stress are recorded. This can be achieved by employing constant strain rate indentation alongside the continuous stiffness method or by utilizing strain rate jump tests [202,203]. The influence of temperature on dislocation mobility and the activation energy necessary for deformation is significant. Increased temperatures typically enhance strain rate sensitivity by promoting more efficient dislocation movement. The microstructure, encompassing aspects such as grain size and dislocation density, plays a crucial role in determining strain rate sensitivity. Enhanced microstructures or elevated dislocation densities may result in heightened sensitivity owing to a greater number of active deformation mechanisms [195,202,203]. The strain rate sensitivity test offers a quantitative assessment of strain rate sensitivity, commonly represented as ‘m,’ which reflects the variation in flow stress of a material in response to different strain rates. A smaller ‘m’ value implies greater resistance to creep deformation, while larger values suggest that the material is more susceptible to deformation [47].

#### 3.2.5. Fatigue and Fracture Toughness Measurements

Fatigue in nanomaterial-based systems typically entails the emergence and expansion of localized shear transformation zones, especially in areas of stress concentration. The presence of these zones results in the collapse and merging of voids, which significantly improves the material’s strain-hardening capacity and engineering stress under cyclic deformation [204]. Cyclic nanoindentation serves as an effective approach for exploring fatigue in nanomaterials, providing valuable insights into their nanomechanical behavior and the mechanisms of failure at the nanoscale. This approach entails subjecting a material to a series of loading and unloading cycles to investigate its fatigue characteristics. Utilizing both high- and low-frequency indentation modes allows for the acquisition of high cycle numbers while simultaneously gathering enough data points to accurately reconstruct force-displacement hysteresis loops. This methodology aids in comprehending the fatigue mechanisms at the nanoscale, as evidenced by research conducted on ductile metals and brittle ceramics [205]. The cyclic nanoindentation technique is instrumental in evaluating how materials respond to cyclic stress, including variations in hardness, elastic modulus, and phase transformations [206,207,208]. Cyclic nanoindentation serves to elucidate plastic deformation behaviors, including strain softening and the development of pileups surrounding the indentation site. The observed phenomena clearly demonstrate the material’s capacity for plastic deformation when subjected to cyclic stress [207,208]. Cyclic nanoindentation in coatings provides insights into low-cycle fatigue through the examination of damage accumulation and the progression of microcracks. Multilayer coatings exhibit enhanced fracture toughness attributed to plastic deformation, as demonstrated by cyclic nanoindentation [209]. Cyclic nanoindentation plays a crucial role in the development of a damage indicator within the context of creep-fatigue interactions. For 316H austenitic stainless steel, a normalized damage indicator, ln(H/E)/m, is proposed to characterize the propagation of trans-granular (fatigue damage) and intergranular (creep damage) cracking in creep-fatigue fracture. This is based on the morphologies of the fracture surface and variations in hardness (H), elastic modulus (E), and strain rate sensitivity (m) [109]. In Mg–SiC nanocomposites, cyclic nanoindentation revealed that increasing reinforcement content led to reduced indentation depth and plastic deformation, indicating enhanced cyclic strength and fatigue resistance [210]. In case of silver/polyvinyl alcohol nanocomposite thin films, cyclic nanoindentation showed linear increases in contact depth and stiffness over cycles, with no evidence of fracture or localized damage, suggesting a uniform and defect-free surface with good fatigue performance [211]. In multilayer and monolayer coatings, such as (TiAlCrSiY)N/(TiAlCr)N, cyclic nanoindentation helped distinguish between microcrack nucleation and microplastic deformation, demonstrating that multilayer structures offer higher fracture toughness due to their ability to accommodate damage through plasticity [209]. Fracture toughness is a critical parameter that assesses a material’s capacity to withstand crack propagation when subjected to stress, effectively quantifying the energy necessary for a crack to advance further. Nanoindentation is widely used to determine the fracture toughness of nanomaterial-based systems by applying a controlled load with a sharp indenter to generate cracks, whose lengths are then measured, often with scanning electron microscopy (SEM), to calculate the critical stress intensity factor (KIc) using models like the Laugier model [212,213]. This technique provides mechanical parameters such as hardness and Young’s modulus alongside fracture toughness, enabling detailed characterization of coatings, thin films, and multiphase layers at micro- and nanoscales [214,215]. Measuring fracture toughness reveals how microstructural features and chemical composition influence mechanical integrity, such as the role of chromium borides in reducing toughness in high-entropy alloys or the effect of sublayers on diamond-like coatings [215,216]. Additionally, nanoindentation combined with imaging and energy-based analysis can elucidate micro-fracture mechanisms, including elastic–plastic deformation and energy dissipation, which are critical for understanding failure modes in brittle nanomaterials [214,217]. Advanced approaches like deep learning applied to nanoindentation images further enhance fracture toughness prediction, offering rapid and detailed insights into complex heterogeneous nanomaterials [218].

#### 3.2.6. Nanoscale Tribological Test

The nanoscratch test has become an essential method for investigating the mechanical and tribological properties of materials, especially thin films and nanoscale structures. It provides important insights into surface integrity, friction, wear resistance, and interfacial adhesion when subjected to controlled mechanical loading conditions [29,219,220,221,222]. A range of methodologies has emerged in the field of nanoscratch testing, designed to replicate different loading scenarios and material degradation processes, with each approach providing distinct benefits tailored to characterization objectives. The ramped load scratch test, commonly known as the progressive scratch test, is a widely utilized method in which the normal load applied to the indenter increases linearly over a specified scratch length or duration [29,219,221,222,223,224,225]. This method is commonly employed to pinpoint the critical loads at which failure events take place, including the initiation of yielding, the elastic–plastic transition, cracking, fragmentation, or delamination [29,43,219,222,224]. The procedure typically entails conducting a pre-scan to determine the initial surface profile prior to scratching, followed by a post-scan to analyze the residual scratch morphology [219,224]. In contrast, the constant load scratch test applies a consistent normal load during the scratching process, which is especially beneficial for examining material behavior and wear progression under a steady stress level [38,49,219,222]. To accurately replicate the fatigue-induced wear that coatings undergo in practical scenarios, the repetitive (cyclic) scratch test applies a subcritical load multiple times along the same scratch track. This method facilitates the investigation of damage accumulation and allows for the assessment of the number of cycles necessary to induce coating failure [225]. Additionally, a new approach referred to as the statistically distributed nanoscratch test entails executing several parallel scratches within a specified region, with the initial positions determined by computer-generated statistical distributions. This technique was designed to more accurately replicate abrasive wear phenomena, where the interplay between damage from neighboring contacts plays a crucial role in material removal. This approach allows the examination of the cycle-by-cycle progression of the abraded area and film degradation by analyzing average properties such as depth and friction [219,224]. Nanoscratching, beyond its role in friction studies, is widely utilized to explore wear mechanisms and material removal processes, enhancing the comprehension of material behavior under conditions of abrasive or sliding contact [43,219,221,224,226]. The evaluation of wear resistance is made possible, often analyzing parameters like specific scratch energy [227] and the progression of damage or transitions between various wear regimes [219,225]. The integration of electrochemical techniques with nanoscratching allows for the evaluation of tribo-corrosion behavior, enabling an assessment of material performance under concurrent mechanical and electrochemical loading in environments such as saline solutions. This approach aids in understanding corrosion resistance in conjunction with abrasion in corrosive media [29]. Nanoscratching offers important insights into the mechanisms of material deformation at micro and nanoscales, highlighting the transitions between elastic, plastic, and brittle-to-ductile fracture behaviors across different loading conditions [31,38,43,228]. The examination of subsurface deformation and defect formation, such as densification, amorphous phases, dislocations, cracks, and stacking faults, also represents a critical focus of research [221,222,223,224,228]. The assessment of interfacial adhesion strength, especially in thin-film/substrate systems, represents a significant application of nanoscratching [29,43,219,222]. The critical load at which adhesion failure occurs serves as a reliable indicator of the strength of this interface [29,219,229]. Friction Force Microscopy (FFM), which is frequently referred to as lateral force microscopy (LFM), is commonly characterized as a variant of the atomic force microscope (AFM). It stands out as a robust tool for examining friction at the nanoscale, especially in the tiniest sliding contacts [230,231,232]. FFM has been utilized to investigate the nanoscale tribological properties of a range of materials and systems, including multilayer graphene, h-BN, and MoS_2_ [233,234,235]. Research has explored surface corrugations such as atomic-scale ripples and nanoscale wrinkles on graphene, revealing that they develop along specific crystallographic directions [233,235]. The long-term wear characteristics of single-layer h-BN, MoS_2_, and graphene were examined through AFM-based wear tests [235]. In this research, Friction Force Microscopy (FFM) was employed as a critical post-mortem diagnostic tool to qualitatively assess the integrity of the two-dimensional (2D) material coatings after long-term wear testing. FFM images of the hexagonal boron nitride (h-BN) wear tracks revealed a distinct region of high friction. This high-friction signal corresponded to the bare silicon dioxide (SiO_2_) substrate, providing clear evidence that the h-BN film had been completely worn away during the test. In stark contrast, the FFM images of MoS_2_ and graphene specimens showed a consistent and uniform low-friction signal across the entire scanned area, including the wear track, even after one million cycles. This result was crucial because while topographic AFM images showed a depression in the surface, it was very hard to distinguish between film wear and plastic deformation of the underlying substrate. The uniform FFM signal demonstrated that the MoS_2_ and graphene layers remained intact, proving their exceptional wear resistance and confirming that the surface depression was due to the deformation of the substrate beneath the protective 2D film. For all three materials, FFM confirmed that the presence of surface wrinkles led to rapid and complete failure. The images clearly showed high-friction wear tracks where the 2D material had been removed from the wrinkled areas. FFM has been employed to explore GeAs nanofilms with different thicknesses, uncovering relationships among friction, layer count, surface potential, and adhesion [236]. A significant insight of this research is the use of FFM in conjunction with Kelvin Probe Force Microscopy (KPFM). This combination allowed researchers to directly link a material’s mechanical (friction, adhesion) and electrical (surface potential) properties. In the study, FFM revealed that friction on GeAs increased as the film thickness increased, a trend opposite to most 2D materials like graphene. By using KPFM analysis on the same area, they discovered that the surface potential also increased with thickness.

#### 3.2.7. Role of Computational Materials Science in Studying Nanomaterial-Based Systems at Nanoscale

Computational materials science has become indispensable for unraveling nanoscale corrosion mechanisms and designing corrosion-resistant nanomaterials. By integrating quantum mechanical, atomistic, and data-driven approaches, researchers can probe corrosion processes at unprecedented spatial and temporal resolutions, enabling both fundamental understanding and accelerated materials discovery. Computational methods such as density functional theory (DFT), molecular dynamics (MD), phase-field modeling, and machine learning (ML) allow for the simulation of atomic-scale interactions, defect evolution, and environmental effects that govern corrosion at the nanoscale. These approaches provide insights into how nanostructures, grain boundaries, and surface chemistry influence corrosion initiation and propagation—phenomena often inaccessible to experimental techniques alone [237,238,239,240]. DFT calculations have been used to predict the adsorption energies and electronic properties of corrosion inhibitors on metal surfaces, guiding the rational design of more effective inhibitors [237,238]. Phase-field models have elucidated how nanoscale precipitates and grain boundary characteristics affect intergranular corrosion in aluminum alloys, revealing that the orientation, size, and distribution of nano-precipitates can dramatically alter corrosion rates and mechanisms [64]. Multiscale simulations, combining ab initio, MD, and continuum models, have clarified the interplay between atomic-scale defects and macroscopic corrosion behavior in nano-coatings [239]. Computational materials science accelerates the discovery and optimization of corrosion-resistant nanomaterials by enabling high-throughput screening and predictive modeling. Machine learning frameworks, trained on experimental and first-principles data, have been developed to identify corrosion-resistant high-entropy alloys and Mg-based intermetallics, significantly reducing the need for costly trial-and-error experiments [241,242]. These models can predict key properties such as surface energy, phase stability, and hydrogen adsorption, which are critical for corrosion resistance. ML-accelerated high-throughput calculations have identified promising binary Mg intermetallics that suppress galvanic corrosion, while random forest models have been used to map the compositional space of high-entropy alloys for optimal corrosion resistance [241,242]. DFT and MD simulations also inform the design of nanostructured coatings and inhibitors by revealing atomic-scale mechanisms of passivation, self-healing, and barrier formation [237,238]. Computational studies guide the design of advanced self-healing anti-corrosion coatings by revealing molecular-level healing mechanisms and enabling the prediction of macroscopic coating performance [53]. Computational materials science has also become indispensable for understanding and optimizing nanoscale mechanical strengthening and tribological wear mechanisms in nanomaterial-based systems. Molecular dynamics (MD), density functional theory (DFT), and multiscale modeling enable the exploration of atomic-level deformation, defect evolution, and load transfer mechanisms that are challenging to capture experimentally. MD simulations have revealed how varying nanoparticle size and volume fraction in metal matrix nanocomposites like Al-SiC influence transitions from defect-free deformation to dislocation-driven and interface-failure mechanisms, directly linking nanoscale features to macroscopic strength and toughness [243,244,245]. Multiscale frameworks bridge atomistic and continuum scales, allowing accurate prediction of nonlinear mechanical behavior in nanocrystalline structures and providing constitutive models for large-scale analysis [244,245,246]. Machine learning models, trained on simulation and experimental data, further accelerate the prediction of mechanical properties, such as elastic modulus and tensile strength, with high accuracy and efficiency [247,248]. Machine learning-augmented multiscale framework accurately predicted the mechanical response of nanocrystalline structures under various deformation paths, validated against both atomistic simulations and experiments [246]. Similarly, hierarchical graph neural networks have enabled rapid, reliable predictions of mechanical properties in carbon nanostructures, even accounting for defects [247]. Computational materials science is equally transformative in tribology, where wear and friction at the nanoscale are governed by complex, multi-parameter interactions. MD simulations and machine learning models can dissect the influence of nanofiller type, dispersion, and interfacial bonding on wear resistance and friction coefficients [116,249,250,251]. These tools allow for the identification and prediction of dominant wear mechanisms—such as abrasion, adhesion, oxidation, and delamination—under varying loads, speeds, and environmental conditions [249,252,253,254]. Machine learning models, including Random Forest and Extreme Gradient Boosting, have been used to predict wear rates and coefficients of friction in nanocomposites, reducing the need for extensive physical testing and enabling rapid optimization of material formulations [249,251,252,254]. MD simulations have elucidated how the dimensionality and functionalization of carbon nanomaterials (e.g., graphene, nanotubes) in polymer matrices dramatically reduce wear and friction by mechanisms such as tribo-film formation and mechanical interlocking [116,250,255].

## 4. Challenges in Characterization of Nanomaterial-Based System Properties

### 4.1. Challenges in Nanoscale Corrosion Measurements

Characterizing the nanoscale corrosion behavior of nanomaterial-based systems poses a range of complex challenges for researchers. These challenges arise primarily from the small dimensions and unique properties of these materials, which frequently display physicochemical characteristics that differ significantly from those of their bulk counterparts, largely due to their considerably higher surface area-to-volume ratio [57,256]. Furthermore, the investigation of localized corrosion, which takes place at specific sites over extremely small length scales, reaching down to nanometers, and rapid time scales down to sub-seconds, presents significant challenges. This is primarily because many traditional characterization methods do not possess the required spatial or temporal resolution for in situ observation of these initial corrosion phenomena [39,140]. Accurately understanding the corrosion mechanism and the heterogeneous dissolution kinetics of metallic materials across a wide range of length scales, from nanometers to micrometers, is both crucial and demanding. It requires methods that are sensitive enough to capture events at the finest resolutions [40]. The characterization of nanomaterials demands high sensitivity and selectivity, alongside precision. Researchers encounter the challenge that there is no universally applicable “best electrochemical technique” for all characterization scenarios. This often necessitates a careful selection process tailored to the specific materials, their intended applications, and the limitations of the techniques at hand. The existing experimental techniques encounter specific challenges when utilized for nanoscale corrosion studies. Scanning Electron Microscopy (SEM) is capable of examining surface features at nanometric resolution, yet it frequently experiences considerable uncertainties in measuring surface roughness, a vital factor in comprehending corrosion morphology [257]. Certain techniques, such as Scanning Electrochemical Cell Microscopy (SECCM), while beneficial for microscopic corrosion measurements, utilize probes that generally range from 0.2 to 50 µm in size. This size is notably larger than the nanoscale resolution that methods like AFM can achieve [40]. While multifrequency atomic force microscopy (AFM) provides essential tools for three-dimensional characterization and nanoscale roughness quantification of micro–nano-plastics (MNPs), it falls short in reliably differentiating between various polymers based on elastic modulus assessments. This limitation stems from errors that occur due to the irregular contact geometry between the AFM probe and the sample surface, which diverges from the idealized models employed for calculations [257]. AFM, despite its capabilities, is noted for having a restricted single-shot scanning area and height range, as well as relatively slow raster speeds, which could result in overlooking dynamic and rapidly changing corrosion events. Additionally, the surface topography obtained from an AFM image may occasionally represent the interaction between the probe and the surface instead of accurately depicting the true surface morphology, which can lead to potential artifacts [40]. Optical techniques, commonly employed in corrosion studies, presently fall short in delivering high-quality quantitative kinetic data or fundamental mechanisms of metallic corrosion at micro- and nanoscales in real-time. Much of the existing research has depended on ex situ investigations, underscoring the necessity for innovative methods to address this gap [40]. Liquid cell Transmission Electron Microscopy (LC-TEM) is a sophisticated method for examining processes in liquid environments, yet it encounters considerable challenges due to the impact of the electron beam. This influence can modify the conditions within the liquid cell, affecting temperature or pH, and may even cause direct damage to the sample being observed. As a result, meticulous parameter adjustments are required, along with complementary ex situ experiments to anticipate and alleviate these effects [147,149]. The accumulation of charge on the insulating components of liquid cells, including acetonitrile or silicon nitride, during electron illumination poses a significant challenge. This phenomenon can adversely affect imaging quality and may result in sample decomposition at elevated irradiation levels, complicating the maintenance of stable observation conditions [147]. In the comparison of advanced microscopy techniques and conventional electrochemical methods for corrosion studies, a significant difference is evident in the observation scale and the nature of the information gathered. Traditional macroscopic or bulk electrochemical techniques, such as potentiodynamic scanning, electrochemical impedance spectroscopy, potentiometric, cyclic voltammetry (CV), linear sweep, and pulsed methods, have established themselves as the benchmark for examining corrosion kinetics and potentials, as illustrated in many studies [23,41,60,258]. Nanocomposite coatings are, by nature, heterogeneous. They contain features such as nanoparticle agglomerates, nanoscale pores, and variations in material density. Conventional electrochemical techniques provide a spatially averaged response over a macroscopic area. This averaging effect obscures the localized electrochemical activity at nanoscale defects, which are the critical initiation sites for pitting and other forms of localized corrosion. The excellent performance of the bulk coating can therefore mask the signature of incipient failure, leading to an overestimation of the coating’s protective capability [40]. Many advanced coatings, particularly those incorporating graphene or exhibiting superhydrophobicity, possess extremely high impedance. This property, while desirable for corrosion protection, complicates electrochemical analysis. The resulting corrosion currents are often vanishingly small, approaching the noise floor of standard potentiostats and leading to a low signal-to-noise ratio [259]. Furthermore, aggressive techniques like potentiodynamic polarization, which apply high potentials to force a measurable current, can introduce irreversible damage to the coating’s delicate nanostructure, causing the test itself to induce failure pathways [260]. The interpretation of electrochemical impedance spectroscopy data relies on fitting the spectra to equivalent electrical circuits that model the physical phenomena at the coating–substrate interface. The complex, multilayered nature of nanocomposite coatings often gives rise to multiple, overlapping time constants that cannot be adequately described by simple EECs. The development of a physically meaningful model is non-trivial, and the use of overly complex circuits with too many variables can lead to ambiguity and scientifically unsound conclusions [261].

### 4.2. Challenges in the Characterization of Nanoscale Mechanical and Tribological Properties

Characterizing the nanomechanical and nanotribological properties of nanomaterial-based systems is essential for comprehending their prospective applications across diverse domains, such as surface engineering, coating industries, biomaterials, and composites. Nevertheless, numerous restrictions must be resolved to improve the precision and dependability of these characterizations. The difficulties in this characterization derive from the distinctive features of nanomaterials and the constraints of existing testing methodologies. Nanomaterials frequently display considerable variability and intricate microstructures that can differ across various lengths. The heterogeneity in nanomaterials manifests as differences in pore form, distribution, and orientation, which profoundly influence their mechanical characteristics, including Young’s modulus [5,262]. Heterogeneity in nanomaterials arises from the incorporation of many phases or components into a singular nanoscale structure. This integration produces varied and adjustable interactions among distinct phases, resulting in novel features unattainable in single-phase materials. Carbon-based heterogeneous structured nanomaterials display unique architecture and intriguing features attributable to the varied bonding capabilities of carbon allotropes [5]. Surface roughness can profoundly influence the mechanical properties of nanomaterials, including hardness and elastic modulus. The morphology of nanoparticles, including spherical or amorphous forms, might affect their mechanical characteristics and the efficacy of characterization methods [263,264,265]. A study conducted by Bendaoued et al. [263] demonstrated that nanoparticles with reduced surface roughness generally have superior mechanical properties. The TiO_2_, Al_2_O_3_, and SiO_2_ nanopowders exhibit low roughness values, with SiO_2_ (Rq = 0.720 nm) noted for its remarkably high specific surface area, a factor that directly affects mechanical properties such as stiffness and strength. The research demonstrates that the sol–gel method employed in the fabrication of these aerogels produces nanopowders characterized by smooth surfaces and elevated Young’s moduli. A smoother surface reduces stress concentration spots, and this uniformity enhances the load-bearing capacity of the nanoparticles. Consequently, a significant association exists between surface roughness and enhanced mechanical qualities such as stiffness and deformation resistance. Agglomeration and dispersion are pivotal features that complicate the evaluation of the nanomechanical properties of nanomaterials [266,267]. This effect is frequently induced by the elevated surface energy of nanoparticles, leading to their aggregation instead of uniform dispersion within the matrix. In graphene–polymer nanocomposites, agglomeration can significantly impede stress transport at the interface, resulting in a decline in mechanical characteristics such as Young’s modulus [266]. In metal/graphene nanocomposites, agglomeration and the development of interphase zones can result in inconsistencies between theoretical predictions and experimental outcomes concerning the material’s strength [267].

Methods like nanoindentation and atomic force microscopy (AFM) have been established to assess mechanical properties, including hardness and elastic modulus, at the nanoscale. Nonetheless, these methodologies must be modified to consider the distinctive characteristics of nanomaterials, including their elevated surface-to-volume ratio and surface effects, which significantly influence mechanical behavior [268,269,270,271]. The nanoindentation system occasionally encounters difficulties related to instruments, necessitating meticulous calibration to guarantee precise load and displacement measurements. The configuration and geometry of the indenter tip are critical determinants affecting the stress distribution during nanoindentation. Diverse tip geometries, including Berkovich, cube-corner, and spherical, can result in discrepancies in stress distribution and fracture morphology. For example, sharper ends, such as cube corners, can enhance shear stress distribution, rendering coatings more susceptible to delamination and influencing the fracture morphology. This may result in crack formation or dislocation accumulation, impacting the material’s nanomechanical properties [272,273]. The radius of the indenter tip is essential for assessing mechanical properties, including Young’s modulus. The precise measurement of the tip radius is crucial, since it directly influences the force–indentation depth curve and the derived mechanical properties [274]. The tip radius significantly influences the spatial resolution of nanoindentation mapping. In metallic glasses, spatial resolution is determined by the normalized indentation spacing in relation to the tip radius, impacting the assessed hardness and elastic modulus [275]. A key problem in nanoindentation precision is the substrate’s impact on the measurements. The substrate can substantially influence the measured mechanical properties, such as hardness and modulus, when indenting thin films or 2D materials, resulting in erroneous outcomes [174,276,277]. The substrate effect is more significant when the tested material is thin relative to the indentation depth. If the indentation depth significantly exceeds the material’s thickness, the substrate’s characteristics will prevail in the nanomechanical assessment. Characterizing the creep behavior of nanomaterials via nanoindentation creep testing also poses numerous obstacles [278,279]. Minnert et al. [278] scrutinized the difficulties associated with nanoindentation creep testing approaches, namely the constant contact pressure approach. This method maintains consistent pressure throughout the test, producing stress exponents analogous to uniaxial testing, which is beneficial for the characterization of nanomaterials. Nonetheless, it underscores limits, like the relaxing of materials under sustained load conditions, which may influence outcomes. The sensitivity to parameters like indentation depth, loading speed, and holding time during the creep phase substantially influences the reported creep behavior. These factors must be meticulously regulated to guarantee precise and consistent outcomes. Advanced statistical techniques, like ANOVA and random forest analysis, are frequently utilized to determine the most significant testing parameters and to link them with the observed mechanical behavior. This method aids in comprehending the intricate interplay between the testing conditions and the material’s response [279].

Nanoscratch testing, although an effective method for characterizing nanotribological features and elucidating wear mechanisms at the nanoscale, has numerous problems. A notable difficulty is the sensitivity of nanoscratching results to testing parameters. The outcome can be significantly affected by parameters like the applied normal force, scratch speed, indenter shape (tip radius, angle) [43,221,222,223,226,227], scratching direction or orientation [223,226,228], loading rate [224,225], and the angle between the indenter edge and scratch direction [227]. In investigations of the Ti–6Al–4V alloy, changes in the angle between the indenter edge and the scratch direction, as well as variations in normal forces and scratch velocities, were seen to affect the coefficient of friction (COF), scratch depth, width, morphology, and specific scratch energy [227]. Interpreting results, such as delamination energy, can be complicated due to significant parameter sensitivity and the absence of a defined parameter selection methodology for nanoscratching [43]. Nanoscratching can initiate material removal methods such as plastic deformation, cutting, micro-fracture, and delamination; however, the specific failure modes and measured parameters can differ significantly based on testing conditions [43,219,224]. In investigations of tungsten–silicon thin films, nanoscratching showed significant sensitivity to scratch loading, and the occurrence of fragmentation events resulted in elevated delamination energy values relative to those obtained from nanoindentation measurements. Various combinations of scratch parameters produced a broad spectrum of delamination energy values, ranging from 13 to 49 J/m^2^. Increased loading rates correlated with elevated critical loads and reduced delamination energies [43]. The impact of substrate and thin-film characteristics on the observed tribological performance presents an additional hurdle, particularly during thin-film testing [219,224,280]. Research on high-entropy alloy thin films on silicon substrates revealed that the substrate’s characteristics, such as hardness and elastic modulus, along with the film’s mechanical properties, affect the critical load for yielding and the resultant failure modes, including fatigue-like phenomena and substrate chipping [219,224]. Sample heterogeneity may also provide difficulties. Nanoscratch testing on adhesive films demonstrated variations in material properties throughout the layer thickness, corresponding with the phenomena of apparent Young’s modulus change [38]. Shales exhibit intricate internal structures, mineral heterogeneity, and localized characteristics, necessitating high spatial resolution techniques such as nanoscratching; nonetheless, interpreting results across diverse mineral compositions can be challenging [49]. Executing accurate and insightful FFM characterization to ascertain the nanotribological properties of nanomaterials presents substantial obstacles that require meticulous experimental design and data analysis [230,233,234]. A significant obstacle is the difficulty in distinguishing growth-induced surface corrugations on materials such as chemical vapor deposited (CVD) graphene from wrinkles that occur during the transfer process, especially when a supporting layer is employed [233]. The intrinsic ambiguity hinders the identification of the principal mechanism responsible for the formation of graphene corrugations during transfer. Researchers have attempted to mitigate this by developing “quasi-free-standing” graphene using weakly interacting layers, such as h-BN, in a wet transfer process to facilitate the release of growth-induced corrugations [230,233]. Maintaining heterogeneous surfaces during FFM tests presents considerable challenges, as it facilitates the examination of friction between distinct materials and mitigates the influence of self-lubrication, wherein atoms may travel between the tip and sample, resulting in a misleading perception of reduced friction [230].

## 5. Conclusions

The comprehensive examination of nanomaterial-based systems (NBS) presented in this review underscores their transformative potential in advancing surface engineering through the integration of nanoscale components. The superior performance of these systems is fundamentally driven by unique nanoscale mechanisms that differ significantly from bulk material behaviors. In the domain of corrosion protection, efficacy is achieved through passive barrier strategies—such as the creation of tortuous diffusion paths by nanofillers and superhydrophobic air-trapping—as well as active protection mechanisms involving stimuli-responsive inhibitor release and self-healing capabilities. Concurrently, mechanical strengthening is governed by phenomena such as grain boundary strengthening (Hall–Petch effect) and confined layer slip in nanolaminates, while tribological resilience is significantly enhanced by the formation of protective tribo-films by 2D nanomaterials, which mitigate wear through lubrication and stress dissipation. A critical finding of this review is that capturing these intricate structure–property relationships necessitates the deployment of high-resolution, in situ characterization techniques. Advanced microscopy methods, including High-Speed Atomic Force Microscopy (HS-AFM) and Liquid Cell Transmission Electron Microscopy (LC-TEM), have proven indispensable for visualizing real-time defect evolution and localized corrosion initiation at the atomic scale. These are effectively complemented by quantitative nanomechanical assessments, such as nanoindentation and nanoscratch testing, which provide localized measurements of hardness, elastic modulus, and interfacial adhesion strength. Furthermore, the integration of computational materials science, particularly through Molecular Dynamics (MD) and Machine Learning (ML), has emerged as a vital tool for modeling atomic-scale interactions and predicting macroscopic performance, thereby accelerating the optimization of complex nanocomposites.

Despite significant progress in the field, this review also underscores the persistent challenges that impede a complete understanding and widespread application of these technologies. The characterization of nanomaterials remains a complex task, fraught with difficulties related to instrumentation limits, sample heterogeneity, substrate interference, and the complex interpretation of data. While powerful, existing techniques often provide an incomplete picture, necessitating the development of more integrated and multi-modal approaches. Looking forward, the future of this field lies in addressing these challenges and exploring new research frontiers. A primary forward-looking agenda must be the standardization of in situ nanomechanical and electrochemical testing. Current methods struggle to decouple substrate effects from thin-film properties during nanoindentation, leading to erroneous modulus and hardness data. Future research should focus on developing universal correction algorithms and standardized probe geometries (e.g., consistent tip radii) to ensure reproducibility across different laboratories. Furthermore, establishing protocols for operando testing—measuring properties while the material is under actual service loads and corrosive conditions—is essential to bridge the gap between laboratory “ideal” behaviors and industrial “real-world” failure. The heterogeneity of nanomaterial systems—where nanoparticle agglomeration, phase boundaries, and defects create complex, nonlinear responses—poses a significant challenge for predictive modeling. The next frontier lies in the integration of artificial intelligence (AI) and machine learning (ML) with multiscale physical modeling to create “Digital Twins” of nanomaterial-based systems. Current computational approaches (DFT, MD) are computationally expensive and limited to small time/length scales. A robust research agenda should prioritize the development of physics-informed neural networks that can learn from sparse experimental data (e.g., from high-throughput nanoindentation mapping) to predict macroscopic failure probabilities. This would allow researchers to virtually screen numerous nanocomposite formulations for optimal tribo-corrosion resistance without the need for exhaustive physical testing, specifically targeting the complex interplay between nanofiller dispersion and localized stress concentration. Finally, environmental regulations are forcing a pivot toward green tribology. The traditional reliance on toxic, oil-based lubricants and heavy-metal coatings is becoming untenable. A rapidly emerging agenda is the enhancement of waste streams—converting agro-industrial residues like rice husks, eggshells, and waste rubber into high-performance nanoparticle additives for lubricants and coatings. These waste-derived nanoparticles not only reduce the carbon footprint of material synthesis but also offer tribological performance comparable to synthetic counterparts. Future research must focus on the long-term stability and dispersion of these green nanomaterials, as well as their end-of-life biodegradability, ensuring that the solution to wear and corrosion does not become a new source of environmental micro-pollution.

## Data Availability

The original contributions presented in the study are included in the article; further inquiries can be directed to the corresponding author.

## References

[1] Bromma K., Alhussan A., Perez M.M., Howard P., Beckham W., Chithrani D.B. (2021). Three-Dimensional Tumor Spheroids as a Tool for Reliable Investigation of Combined Gold Nanoparticle and Docetaxel Treatment. Cancers.

[2] Bromma K., Dos Santos N., Barta I., Alexander A., Beckham W., Krishnan S., Chithrani D.B. (2022). Enhancing Nanoparticle Accumulation in Two Dimensional, Three Dimensional, and Xenograft Mouse Cancer Cell Models in the Presence of Docetaxel. Sci. Rep..

[3] Harish V., Ansari M.M., Tewari D., Gaur M., Yadav A.B., García-Betancourt M.-L., Abdel-Haleem F.M., Bechelany M., Barhoum A. (2022). Nanoparticle and Nanostructure Synthesis and Controlled Growth Methods. Nanomaterials.

[4] Kang J., Yang X., Hu Q., Cai Z., Liu L.-M., Guo L. (2023). Recent Progress of Amorphous Nanomaterials. Chem. Rev..

[5] Yin Y., Hou X., Wu B., Dong J., Yao M. (2024). Heterogeneous Structured Nanomaterials from Carbon and Related Materials. Adv. Funct. Mater..

[6] Mekuye B., Abera B. (2023). Nanomaterials: An Overview of Synthesis, Classification, Characterization, and Applications. Nano Sel..

[7] Al-Harbi N., Abd-Elrahman N.K. (2024). Physical Methods for Preparation of Nanomaterials, Their Characterization and Applications: A Review. J. Umm Al Qura Univ. Appl. Sci..

[8] Paras, Yadav K., Kumar P., Teja D.R., Chakraborty S., Chakraborty M., Mohapatra S.S., Sahoo A., Chou M.M.C., Liang C.-T. (2022). A Review on Low-Dimensional Nanomaterials: Nanofabrication, Characterization and Applications. Nanomaterials.

[9] Zhao C., Kang J., Li Y., Wang Y., Tang X., Jiang Z. (2023). Carbon-Based Stimuli-Responsive Nanomaterials: Classification and Application. Cyborg Bionic Syst..

[10] Rozhdestvenskiy O.I., Lalitha Y.S., Ikram M., Gupta M., Jain A., Verma R., Sood S. (2024). Advances in Nanomaterials: Types, Synthesis, and Manufacturing Methods. E3S Web Conf..

[11] Rizwan M., Shoukat A., Ayub A., Razzaq B., Tahir M.B. (2021). Types and Classification of Nanomaterials. Nanomaterials: Synthesis, Characterization, Hazards and Safety.

[12] Harish V., Tewari D., Gaur M., Yadav A.B., Swaroop S., Bechelany M., Barhoum A. (2022). Review on Nanoparticles and Nanostructured Materials: Bioimaging, Biosensing, Drug Delivery, Tissue Engineering, Antimicrobial, and Agro-Food Applications. Nanomaterials.

[13] Saleh H.M., Hassan A.I. (2023). Synthesis and Characterization of Nanomaterials for Application in Cost-Effective Electrochemical Devices. Sustainability.

[14] Mohammed H., Mia M.F., Wiggins J., Desai S. (2025). Nanomaterials for Energy Storage Systems—A Review. Molecules.

[15] Osman A.I., Zhang Y., Farghali M., Rashwan A.K., Eltaweil A.S., Abd El-Monaem E.M., Mohamed I.M.A., Badr M.M., Ihara I., Rooney D.W. (2024). Synthesis of Green Nanoparticles for Energy, Biomedical, Environmental, Agricultural, and Food Applications: A Review. Environ. Chem. Lett..

[16] Darwish M.A., Abd-Elaziem W., Elsheikh A., Zayed A.A. (2024). Advancements in Nanomaterials for Nanosensors: A Comprehensive Review. Nanoscale Adv..

[17] Ramteke S.M., Walczak M., Stefano M.D., Ruggiero A., Rosenkranz A., Marian M. (2024). 2D Materials for Tribo-Corrosion and -Oxidation Protection: A Review. Adv. Colloid Interface Sci..

[18] AhadiParsa M., Dehghani A., Ramezanzadeh M., Ramezanzadeh B. (2022). Rising of MXenes: Novel 2D-Functionalized Nanomaterials as a New Milestone in Corrosion Science—A Critical Review. Adv. Colloid Interface Sci..

[19] Nazari M.H., Zhang Y., Mahmoodi A., Xu G., Yu J., Wu J., Shi X. (2022). Nanocomposite Organic Coatings for Corrosion Protection of Metals: A Review of Recent Advances. Prog. Org. Coat..

[20] Nabhan F., Fayyad E.M., Sliem M.H., Shurrab F.M., Eid K., Nasrallah G., Abdullah A.M. (2023). ZnO-Doped gC_3_N_4_ Nanocapsules for Enhancing the Performance of Electroless NiP Coating—Mechanical, Corrosion Protection, and Antibacterial Properties. ACS Omega.

[21] Verma C., Berdimurodov E., Verma D.K., Berdimuradov K., Alfantazi A., Hussain C.M. (2023). 3D Nanomaterials: The Future of Industrial, Biological, and Environmental Applications. Inorg. Chem. Commun..

[22] Yadav S., Raman A.P.S., Singh M.B., Massey I., Singh P., Verma C., AlFantazi A. (2024). Green Nanoparticles for Advanced Corrosion Protection: Current Perspectives and Future Prospects. Appl. Surf. Sci. Adv..

[23] Rai T.Y., Hegde A.C. (2025). Electrochemical Development and Characterisation of Nanostructured Ni–Fe Alloy Coatings for Corrosion Protection. Can. Metall. Q..

[24] Piscitelli F., Volpe A. (2024). Superhydrophobic Coatings for Corrosion Protection of Stainless Steel. Aerospace.

[25] Yao C.-W., Lian I., Zhou J., Bernazzani P., Jao M., Hoque M.A. (2025). Corrosion Resistance and Nano-Mechanical Properties of a Superhydrophobic Surface. Lubricants.

[26] Muresan L.M. (2023). Nanocomposite Coatings for Anti-Corrosion Properties of Metallic Substrates. Materials.

[27] Farooq S.A., Raina A., Mohan S., Singh R.A., Jayalakshmi S., Haq M.I.U. (2022). Nanostructured Coatings: Review on Processing Techniques, Corrosion Behaviour and Tribological Performance. Nanomaterials.

[28] Deyab M.A., Alghamdi M.M., El-Zahhar A.A., El-Shamy O.A.A. (2024). Advantages of CoS_2_ Nano-Particles on the Corrosion Resistance and Adhesiveness of Epoxy Coatings. Sci. Rep..

[29] Hoque M.A., Yao C.-W., Khanal M., Lian I. (2022). Tribocorrosion Behavior of Micro/Nanoscale Surface Coatings. Sensors.

[30] Wang R., Cao L., Wang W., Mao Z., Han D., Pei Y., Chen Y., Fan W., Li W., Chen S. (2024). Construction of Smart Coatings Containing Core-Shell Nanofibers with Self-Healing and Active Corrosion Protection. ACS Appl. Mater. Interfaces.

[31] Sadeghi B., Cavaliere P., Shabani A., Pruncu C.I., Lamberti L. (2024). Nano-Scale Wear: A Critical Review on Its Measuring Methods and Parameters Affecting Nano-Tribology. Proc. Inst. Mech. Eng. Part J J. Eng. Tribol..

[32] Fan X., Xing Y., Wu Z., Li B., Huang P., Liu L. (2024). Controllable Interface-Tailored Strategy to Reduce the Nanotribological Properties of Ti_3_C_2_T*_x_* by Depositing MoS_2_ Using Atomic Layer Deposition. Nanotechnology.

[33] Li Y., Wei C., Kooi S.E., Veysset D., Guo C., Gan Y., Zhuo Y., Chen G., Naraghi M., Nelson K.A. (2024). Tough Monolayer Silver Nanowire-Reinforced Double-Layer Graphene. ACS Appl. Mater. Interfaces.

[34] Khan M.S., Katiyar L.K., Sasikumar C. (2025). Influence of Radial Distance from Plasma Stealth Center on Nano-Mechanical and Hydrophobic Characteristics of Diamond-like Carbon/Cu Composite Films Synthesized via PECVD. Diam. Relat. Mater..

[35] Narayana T., Saleem S.S. (2024). Comparative Investigation and Characterization of the Nano-Mechanical and Tribological Behavior of RF Magnetron Sputtered TiN, CrN, and TiB_2_ Coating on Ti6Al4V Alloy. Tribol. Int..

[36] Tai C.-L., You J.-D., Chen J.-J., Liang S.-C., Chung T.-F., Yang Y.-L., Ii S., Ohmura T., Zheng X., Chen C.-Y. (2025). In-Situ Transmission Electron Microscopy Investigation of the Deformation Mechanism in CoCrNi and CoCrNiSi_0.3_ Nanopillars. Scr. Mater..

[37] Fard M.Y., Norkus T. (2024). Introducing a Nano-Scale Surface Morphology Parameter Affecting Fracture Properties of CNT Nanocomposites. Compos. Commun..

[38] Anasiewicz K. (2024). Studies of Nanoscratching in the Aspect of Homogeneity of Adhesive Joints. Manuf. Technol..

[39] Peng X., Shangguan J., Zhang Q., Hauwiller M., Yu H., Nie Y., Bustillo K.C., Alivisatos A.P., Asta M., Zheng H. (2024). Unveiling Corrosion Pathways of Sn Nanocrystals through High-Resolution Liquid Cell Electron Microscopy. Nano Lett..

[40] Fanijo E.O., Thomas J.G., Zhu Y., Cai W., Brand A.S. (2022). Surface Characterization Techniques: A Systematic Review of Their Principles, Applications, and Perspectives in Corrosion Studies. J. Electrochem. Soc..

[41] Xavier J.R., Vinodhini S.P., Shanmuga Sundari C., Dhanalakshmi C., Raja Beryl J. (2023). Electrochemical Characterizations of the Anticorrosive Nanoscale Polymer-Based Coatings. Polymer-Based Nanoscale Materials for Surface Coatings.

[42] Feng K.-X., Lai T., Chen Y., Yin Z., Wu Z.-Q., Yan H., Song H.-G., Luo C., Hu Z. (2024). Micro-Galvanic Corrosion Behaviour of Mg−(7,9)Al−1Fe−xNd Alloys. Trans. Nonferrous Met. Soc. China.

[43] Almarri S., Lloyd M., Darnbrough E., Armstrong D. (2025). Quantifying Delamination Energy in Tungsten on Silicon Thin Films through Nanoindentation and Nanoscratch. Mater. Des..

[44] Zhang P., Zhang D., Zhao J. (2024). Control of Fracture Toughness of Kerogen on Artificially-Matured Shale Samples: An Energy-Based Nanoindentation Analysis. Gas Sci. Eng..

[45] Bavdekar S., Rudawski N.G., Basu S., Subhash G. (2024). Microstructural Characterization and Nanomechanical Properties of Multilayer Graphene on Metal Substrates. JOM.

[46] Brizuela-Colmenares N., Pérez-Andrade L.I., Perez S., Brewer L.N., Muñoz-Saldaña J. (2024). Mechanical and Microstructural Behavior of Inconel 625 Deposits by High-Pressure Cold Spray and Laser Assisted Cold Spray. Surf. Coat. Technol..

[47] Wang J., Yang C., Liu Y., Li Y., Xiong Y. (2022). Using Nanoindentation to Characterize the Mechanical and Creep Properties of Shale: Load and Loading Strain Rate Effects. ACS Omega.

[48] Habib S.M.Y., Fan Z., Wang K., Yan Y., Badhan N.A., Fan X. (2025). Effects of Al Addition on the Mechanical and Corrosion Properties of NbTaTiV High-Entropy Alloy. J. Alloys Compd..

[49] Yang L., Yang D., Zhang M., Meng S., Wang S., Su Y., Long X. (2024). Application of Nano-Scratch Technology to Identify Continental Shale Mineral Composition and Distribution Length of Bedding Interfacial Transition Zone—A Case Study of Cretaceous Qingshankou Formation in Gulong Depression, Songliao Basin, NE China. Geoenergy Sci. Eng..

[50] Zhang X., Zheng P., Ma Y., Jiang Y., Li H. (2022). Atomic-Scale Understanding of Oxidation Mechanisms of Materials by Computational Approaches: A Review. Mater. Des..

[51] Dong C., Ji Y., Wei X., Xu A., Chen D., Li N., Kong D., Luo X., Xiao K., Li X. (2021). Integrated Computation of Corrosion: Modelling, Simulation and Applications. Corros. Commun..

[52] Li X., Rong J., Bu J., Sui Y., Zhang Y., Wei Y. (2024). Electrochemical Model of Anodic Dissolution for Magnesium Nanoparticles. Ionics.

[53] Huang L., Chen W., Hao W., Wang J., Guo X., Ma L., Zhang D. (2024). Self-healing Anti-corrosion Coatings: A Mechanism Study Using Computational Materials Science. Electr. Mater. Appl..

[54] Zhang W., Sun J., Ding D., Hou D. (2023). Nanoscale Insights on the Stress Corrosion Mechanism of Calcium-Silicate-Hydrate. J. Build. Eng..

[55] Sun K., Yu S., Yao L., Xu Y. (2025). Atomic-Scale Investigation of the Tribological Behaviors of Titanium Alloy Interfaces with 2D Nanomaterials. Langmuir.

[56] Deka S., Mozafari F., Mallick A. (2023). Microstructural, Mechanical, Tribological, and Corrosion Behavior of Ultrafine Bio-Degradable Mg/CeO_2_ Nanocomposites: Machine Learning-Based Modeling and Experiment. Tribol. Int..

[57] Mofokeng T.G., Motloung M.P., Ama O.M., Ray S.S. (2022). Electrochemical Characterization of Nanomaterials. Characterization of Nanomaterials.

[58] Gao P., Han S., Zhang Y., Fang B., Zhang X., Wang X., Ding H., Zheng K., Pan F. (2024). Investigation on the Interface Bonding and Reinforcement Mechanism of Nano Ti/AZ31 Magnesium Matrix Composites. J. Mater. Res. Technol..

[59] Yamamoto S., Taniguchi D., Okamoto T., Hirata K., Ozawa T., Fukuma T. (2023). Nanoscale Corrosion Mechanism at Grain Boundaries of the Al–Zn–Mg Alloy Investigated by Open-Loop Electric Potential Microscopy. J. Phys. Chem. C.

[60] Pan S., Dong S., Xu M. (2022). Electrochemical Origin for Mitigated Pitting Initiation in AA7075 Alloy with TiB_2_ Nanoparticles. Appl. Surf. Sci..

[61] Lee S., Song G., Yun B., Kim T., Choi S.H., Kim H., Doo S.W., Lee K.T. (2024). Revealing the Nanoscopic Corrosive Degradation Mechanism of Nickel-Rich Layered Oxide Cathodes at Low State-of-Charge Levels: Corrosion Cracking and Pitting. ACS Nano.

[62] Shi Y., Collins L., Balke N., Liaw P.K., Yang B. (2018). In-Situ Electrochemical-AFM Study of Localized Corrosion of Al CoCrFeNi High-Entropy Alloys in Chloride Solution. Appl. Surf. Sci..

[63] Qiu Y., Liu R., Zou L., Chi H., Wang C., Wang B., Chen J. (2022). Influence of Grain Boundary Precipitates on Intergranular Corrosion Behavior of 7050 Al Alloys. Coatings.

[64] Chen Q., Li Z., Yin X., Tang S., Liu W., Ma Y. (2024). Phase-Field Investigation of Intergranular Corrosion Mechanism and Kinetics in Aluminum Alloys. J. Mater. Res. Technol..

[65] Fu X., Deng R., Kong X., Parande G., Hu J., Peng P., Zhu Z., Shi B., Wang G., Gupta M. (2022). Interfacial Characterization and Its Influence on the Corrosion Behavior of Mg-SiO_2_ Nanocomposites. Acta Mater..

[66] Inagaki M., Nishimoto M., Takaya M., Muto I. (2025). In Situ Microscopy and Electrochemical Characteristics of Initiation of Intergranular Corrosion of Aging-Treated Al–1Mg–0.65Si–0.8Cu Alloy in 0.1 M NaCl. Corros. Sci..

[67] Xiao X., Zhou Z., Liu C., Cao L. (2022). Microstructure and Its Effect on the Intergranular Corrosion Properties of 2024-T3 Aluminum Alloy. Crystals.

[68] Bettayeb M., Maurice V., Klein L.H., Lapeire L., Verbeken K., Marcus P. (2018). Nanoscale Intergranular Corrosion and Relation with Grain Boundary Character as Studied In Situ on Copper. J. Electrochem. Soc..

[69] Okonkwo B.O., Li Z., Li L., Chen Y., Li J., You W., Zhang Z., Wang J., Han E.-H. (2025). Multifaceted Study of the Galvanic Corrosion Behaviour of Titanium-TC4 and 304 Stainless Steel Dissimilar Metals Couple in Deep-Sea Environment. Electrochim. Acta.

[70] Cheng T., Huang H., Huang G. (2022). Galvanic Corrosion Behavior between ADC12 Aluminum Alloy and Copper in 3.5 Wt% NaCl Solution. J. Electroanal. Chem..

[71] Liang J., Liu S., Peng Z., Li R., Wang B. (2023). Galvanic Corrosion Behavior of AZ31 Mg Alloy Coupled with Mild Steel: Effect of Coatings. J. Mater. Res. Technol..

[72] Ndukwe A.I., Nwadirichi B., Okolo C., Tom-Okoro M., Medupin R., Uche R., Arukalam I., Onuoha C., Egole C., Okorafor O. (2025). Corrosion Control in Metals: A Review on Sustainable Approach Using Nanotechnology. Zastita Mater..

[73] Brindha T., Rathinam R., Dheenadhayalan S., Sivakumar R. (2021). Nanocomposite Coatings in Corrosion Protection Applications: An Overview. Orient. J. Chem..

[74] Kaya O., Gabatel L., Bellani S., Barberis F., Bonaccorso F., Cole I., Roche S. (2025). 2D Hexagonal Boron Nitride-Based Anticorrosion Coatings. J. Phys. Mater..

[75] Kurtela M., Samardžija M., Bujak M., Stojanović I., Bojanić K., Ljubek G. (2025). Investigation of Corrosion Resistance of Epoxy Coatings Reinforced with Graphene Oxide and Aluminium Nanoparticle. Rud. Geol. Naft. Zb..

[76] Sun T.-Y., Hao Y., Wu Y.-H., Zhao W.-J., Huang L.-F. (2021). Corrosion Resistance of Ultrathin Two-Dimensional Coatings: First-Principles Calculations towards In-Depth Mechanism Understanding and Precise Material Design. Metals.

[77] Hoque M.A., Yao C.-W., Lian I., Zhou J., Jao M., Huang Y.-C. (2022). Enhancement of Corrosion Resistance of a Hot-Dip Galvanized Steel by Superhydrophobic Top Coating. MRS Commun..

[78] Ghaderi M., Bi H., Dam-Johansen K. (2024). Advanced Materials for Smart Protective Coatings: Unleashing the Potential of Metal/Covalent Organic Frameworks, 2D Nanomaterials and Carbonaceous Structures. Adv. Colloid Interface Sci..

[79] Kumar S.S., Kakooei S. (2020). Container-Based Smart Nanocoatings for Corrosion Protection. Corrosion Protection at the Nanoscale.

[80] Raja P.B., Assad M.A., Ismail M. (2020). Inhibitor-Encapsulated Smart Nanocontainers for the Controlled Release of Corrosion Inhibitors. Corrosion Protection at the Nanoscale.

[81] Qian B., Zheng Z., Michailids M., Fleck N., Bilton M., Song Y., Li G., Shchukin D. (2019). Mussel-Inspired Self-Healing Coatings Based on Polydopamine-Coated Nanocontainers for Corrosion Protection. ACS Appl. Mater. Interfaces.

[82] Sanyal S., Park S., Chelliah R., Yeon S.-J., Barathikannan K., Vijayalakshmi S., Jeong Y.-J., Rubab M., Oh D.H. (2024). Emerging Trends in Smart Self-Healing Coatings: A Focus on Micro/Nanocontainer Technologies for Enhanced Corrosion Protection. Coatings.

[83] Xu Y., Liu R., Shao Z., Chen L., Wei W., Dong S., Wang H. (2024). Synergistic Effect of 8-HQ@CeO_2_ for Enhanced Corrosion Resistance of Self-Healing Polyurethane Coating for Corrosion Protection of Mild Steel. Prog. Org. Coat..

[84] González E., Stuhr R., Vega J.M., García-lecina E., Grande H.J., Leiza J.R., Paulis M. (2021). Assessing the Effect of CeO_2_ Nanoparticles as Corrosion Inhibitor in Hybrid Biobased Waterborne Acrylic Direct to Metal Coating Binders. Polymers.

[85] Liu X., Wu Z., Lyu Y., Li T., Yang H., Liu Y., Liu R., Xie X., Lyu K., Shah S.P. (2023). Corrosion Resistance of CeO_2_-GO/Epoxy Nanocomposite Coating in Simulated Seawater and Concrete Pore Solutions. Polymers.

[86] Ma J., Zhang K., Du L., Wang X., Chen Z., Chen H., Chen C., Qiu P. (2024). Intrinsic Self-Healable, Corrosion-Resistant Silicone Coating Based on Quadruple Hydrogen-Bonded Supramolecular Polymer. ACS Appl. Mater. Interfaces.

[87] Gharieh A., Sharifian A., Dadkhah S. (2025). Enhanced Long-Term Corrosion Resistance and Self-Healing of Epoxy Coating with HQ-Zn-PA Nanocomposite. Sci. Rep..

[88] Carneiro Í., Simões S. (2021). Strengthening Mechanisms in Carbon Nanotubes Reinforced Metal Matrix Composites: A Review. Metals.

[89] Pinate S., Ghassemali E., Zanella C. (2022). Strengthening Mechanisms and Wear Behavior of Electrodeposited Ni–SiC Nanocomposite Coatings. J. Mater. Sci..

[90] Lu X., Zhang W., Guo X., Yang X., Li J., Ren J., Xue H., Tang F. (2023). Strengthening Mechanism of NiCoAl Alloy Induced by Nanotwin under Hall-Petch Effect. Int. J. Mech. Sci..

[91] Xie Q., Liu W., Yan X., Zheng H., Li Z., Bai P., Ren L. (2025). Preparation and Mechanical Behavior of Silver-Coated Graphene Reinforced Low Modulus Titanium Matrix Composites by Selective Laser Melting. Mater. Sci. Eng. A.

[92] Lu X., Zhang W., Ren J., Gao Q., Xue H., Tang F., La P., Guo X. (2023). Grain Boundary Segregation Strengthening Behavior Caused by Carbon Chain Network Formation in Nanocrystalline NiCoAl Alloy. J. Mater. Res. Technol..

[93] Usmanov E.I., Gutkin M.Y., Wu Y., Sha G., Valiev R.Z. (2025). Superstrength of Nanostructured Ti Grade 4 with Grain Boundary Segregations. Metals.

[94] Qian L., Zhang J., Yang W., Wang Y., Chan K., Yang X.-S. (2025). Maintaining Grain Boundary Segregation-Induced Strengthening Effect in Extremely Fine Nanograined Metals. Nano Lett..

[95] Lin H., Hua P., Huang K., Li Q., Sun Q. (2023). Grain Boundary and Dislocation Strengthening of Nanocrystalline NiTi for Stable Elastocaloric Cooling. Scr. Mater..

[96] Burtscher M., Kainz C., Dorner P., Fellner S., Terziyska V., Alfreider M., Kiener D. (2025). Phase Stability and Enhanced Mechanical Properties of Nanocrystalline PVD CrCu Coatings. J. Mater. Res. Technol..

[97] Chen Y., Wang Y., Li J. (2024). Size-Dependent Atomic Strain Localization Mechanism in Nb/Amorphous CuNb Nanolayered Composites. J. Appl. Phys..

[98] Shen S., Li H., Wang C., Liang Y., Feng N., Zhang N., Yang L. (2023). Mechanical Properties and Strengthening Mechanism of Ni/Al Nanolaminates: Role of Dislocation Strengthening and Constraint in Soft Layers. Mater. Des..

[99] Nasim M., Li Y., Wen M., Wen C. (2024). Microstructures and Nanomechanical Properties of Nanolaminated Ta/Co Composites and Their Strengthening Mechanisms. Adv. Nanocomposites.

[100] Jian W.-R., Xu S., Su Y., Beyerlein I.J. (2022). Role of Layer Thickness and Dislocation Distribution in Confined Layer Slip in Nanolaminated Nb. Int. J. Plast..

[101] Zhou X., Chen C., Li X. (2025). Layer-Thickness-Dependent Strengthening–Toughening Mechanisms in Crystalline/Amorphous Nanolaminates. ACS Appl. Mater. Interfaces.

[102] Chen X., Fu K., Li Y. (2024). A Theoretical Modelling of Strengthening Mechanism in Graphene-Metal Nanolayered Composites. Int. J. Eng. Sci..

[103] Shi J., Li L., Wang J., Shi T., Zhang Y., Chen J., Cao T., Xu S., Fan X. (2023). Alloy Strengthening toward Improving Mechanical and Tribological Performances of CuxNi100−x/Ta Nano Multilayer Materials. Tribol. Int..

[104] Li C.-L. (2024). Effects of Nd and B Contents on Property Evaluation of (CoCrNi)100–B Nd Medium Entropy Alloy Films. Surf. Coat. Technol..

[105] Xu X., Qiu Z., Zhong J., Tang Y., Zhang L., Ding J., Xia X. (2025). Solid Solution Strengthening and Diffusion Behaviors in Mg–Bi and Mg–Al–Bi Alloys via High-Throughput Measurements. J. Mater. Res. Technol..

[106] Lin D., Motlag M., Saei M., Jin S., Rahimi R.M., Bahr D., Cheng G.J. (2018). Shock Engineering the Additive Manufactured Graphene-Metal Nanocomposite with High Density Nanotwins and Dislocations for Ultra-Stable Mechanical Properties. Acta Mater..

[107] Mohammed O.R., Khan S.M., Patil D.K.A., Chin J. (2025). Wear Resistance and Mechanical Properties of Nanocomposite Coatings: Applications in Aerospace Engineering. Nanotechnol. Percept..

[108] Ghahremani A., Abdullah A., Fallahi Arezoodar A. (2022). Wear Behavior of Metal Matrix Nanocomposites. Ceram. Int..

[109] Banerjee S., Sahoo P. (2022). Fabrication and Investigation of Abrasive Wear Behavior of AZ31-WC-Graphite Hybrid Nanocomposites. Metals.

[110] Rahman E., BinAhmed S., Keyes P., Alberg C., Godfreey-Igwe S., Haugstad G., Xiong B. (2024). Nanoscale Abrasive Wear of Polyethylene: A Novel Approach To Probe Nanoplastic Release at the Single Asperity Level. Environ. Sci. Technol..

[111] Skobeleva N.S., Parfenov A.S., Kopyshev I.Y., Volkov A.V., Berezina E.V. (2023). Mechanism of Abrasion Reduction in Polydisperse System with Carbon Nanoparticles. E3S Web Conf..

[112] Guimarey M.J.G., Ratwani C.R., Xie K., Koohgilani M., Hadfield M., Kamali A.R., Abdelkader A.M. (2023). Multifunctional Steel Surface through the Treatment with Graphene and H-BN. Tribol. Int..

[113] Wang R., Zhang F., Yang K., Xiong Y., Tang J., Chen H., Duan M., Li Z., Zhang H., Xiong B. (2023). Review of Two-Dimensional Nanomaterials in Tribology: Recent Developments, Challenges and Prospects. Adv. Colloid Interface Sci..

[114] Pan J., Zhang R., Yao J., Mu Z., Gu X., Xie Y., Zhang B., Wen M., Liu C., Zhang K. (2025). Contrarian Design of Versatile MoS_2_-Based Self-Lubrication Film. Small.

[115] Zhou S., Wang F., Chen J., Alhashmialameer D., Wang S., Mahmoud M.H.H., Mersal G.A.M., Huang J., Zhang Q., Zhao G. (2022). Enhanced Mechanical, Thermal, and Tribological Performance of 2D-Laminated Molybdenum Disulfide/RGO Nanohybrid Filling Phenolic Resin Composites. Adv. Compos. Hybrid Mater..

[116] Ain Q.U., Wani M.F., Sehgal R., Singh M.K. (2024). Tribological and Mechanical Characterization of Carbon-Nanostructures Based PEEK Nanocomposites under Extreme Conditions for Advanced Bearings: A Molecular Dynamics Study. Tribol. Int..

[117] Vattikuti S.V.P., Byon C. (2015). Synthesis and Characterization of Molybdenum Disulfide Nanoflowers and Nanosheets: Nanotribology. J. Nanomater..

[118] Bobbitt N.S., Curry J.F., Babuska T.F., Chandross M. (2024). Water Adsorption on MoS_2_ under Realistic Atmosphere Conditions and Impacts on Tribology. RSC Adv..

[119] Bondarev A., Ponomarev I., Muydinov R., Polcar T. (2022). Friend or Foe? Revising the Role of Oxygen in the Tribological Performance of Solid Lubricant MoS_2_. ACS Appl. Mater. Interfaces.

[120] Fan X., Shi Y., Cui M., Ren S., Wang H., Pu J. (2021). MoS_2_/WS_2_ Nanosheet-Based Composite Films Irradiated by Atomic Oxygen: Implications for Lubrication in Space. ACS Appl. Nano Mater..

[121] Babuska T.F., Curry J.F., Thorpe R., Chowdhury M.I., Strandwitz N.C., Krick B.A. (2023). High-Sensitivity Low-Energy Ion Spectroscopy with Sub-Nanometer Depth Resolution Reveals Oxidation Resistance of MoS_2_ Increases with Film Density and Shear-Induced Nanostructural Modifications of the Surface. ACS Appl. Nano Mater..

[122] Fu Y., Li H., Chen J., Guo H., Wang X. (2022). Microstructure, Mechanical and Tribological Properties of Arc Ion Plating NbN-Based Nanocomposite Films. Nanomaterials.

[123] Yang Y., Zhou X., Dai X.Z., Li J., Zhang S.H., Zhang C.S., Ding J.C., Zheng J. (2021). Comparative Study of Plasma Nitriding and Plasma Oxynitriding for Optimal Wear and Corrosion Resistance: Influences of Gas Composition. J. Mater. Res. Technol..

[124] Pérez H., Vargas G., Magdaleno C., Silva R. (2023). Article: Oxy-Nitriding AISI 304 Stainless Steel by Plasma Electrolytic Surface Saturation to Increase Wear Resistance. Metals.

[125] Grützmacher P.G., Suarez S., Tolosa A., Gachot C., Song G., Wang B., Presser V., Mücklich F., Anasori B., Rosenkranz A. (2021). Superior Wear-Resistance of Ti_3_C_2_T*_x_* Multilayer Coatings. ACS Nano.

[126] Kumar S., Divakaran A., Kailas S.V. (2023). Fabrication and Tribo Characteristics of In-Situ Polymer-Derived Nano-Ceramic Composites of Al-Mg-Si Alloy. Tribol. Int..

[127] Chen X., Kong Y., Wang M., Huang X., Huang Y., Lv Y., Li G. (2022). Wear and Aging Behavior of Vulcanized Natural Rubber Nanocomposites under High-Speed and High-Load Sliding Wear Conditions. Wear.

[128] Zaharin H.A., Ghazali M.J., Thachnatharen N., Ezzah F., Walvekar R., Khalid M. (2023). Progress in 2D Materials Based Nanolubricants: A Review. FlatChem.

[129] Jiang Y., Turner K.T. (2024). Thermal and Mechanical Mechanisms of Polymer Wear at the Nanoscale. ACS Appl. Mater. Interfaces.

[130] Dang S., Xiao C., Zhang X., Li J., Wang Y., Qian L., Chen L. (2025). Asynchronous Mechanochemical Atomic Attrition Behavior of Heterogeneous Polysilicon Surface. Wear.

[131] Leriche C., Pedretti E., Kang D., Righi M.C., Weber B. (2025). Passivation Species Suppress Atom-by-Atom Wear of Micro-Crystalline Diamond. ACS Appl. Mater. Interfaces.

[132] Tang C., Jiang Y., Chen C., Xiao C., Sun J., Qian L., Chen L. (2024). Graphene Failure under MPa: Nanowear of Step Edges Initiated by Interfacial Mechanochemical Reactions. Nano Lett..

[133] Liu Y., Yang Y., Liu X., Zheng J., Zhang S. (2024). Tribocorrosion of CrN Coatings on Different Steel Substrates. Surf. Coat. Technol..

[134] Fu Y., Zhou F., Wang Q., Zhang M., Zhou Z. (2020). Electrochemical and Tribocorrosion Performances of CrMoSiCN Coating on Ti-6Al-4V Titanium Alloy in Artificial Seawater. Corros. Sci..

[135] Wang Y., Zhang J., Zhou S., Wang Y., Wang C., Wang Y., Sui Y., Lan J., Xue Q. (2020). Improvement in the Tribocorrosion Performance of CrCN Coating by Multilayered Design for Marine Protective Application. Appl. Surf. Sci..

[136] Pramod R., Veeresh K., Basavarajappa S. (2024). Investigation of the Effect of Drilling Induced Delamination and Tool Wear on Residual Strength in Polymer Nanocomposites. FME Trans..

[137] George S.M., Gacem A., Kistan A., Ashick R.M., Rao L.M., Rajput V.S., Nagabooshanam N., Refat M.S., Alsuhaibani A.M., Christopher D. (2022). Investigation of High-Temperature Wear Behaviour of AA 2618-Nano Si_3_N_4_ Composites Using Statistical Techniques. J. Nanomater..

[138] Wei M., Zhang Y., Wang Y., Liu X., Li X., Zheng X. (2024). Employing Atomic Force Microscopy (AFM) for Microscale Investigation of Interfaces and Interactions in Membrane Fouling Processes: New Perspectives and Prospects. Membranes.

[139] Moore S., Burrows R., Kumar D., Kloucek M.B., Warren A.D., Flewitt P.E.J., Picco L., Payton O.D., Martin T.L. (2021). Observation of Stress Corrosion Cracking Using Real-Time in Situ High-Speed Atomic Force Microscopy and Correlative Techniques. npj Mater. Degrad..

[140] Moore S., Burrows R., Picco L., Payton O.D., Martin T.L. (2022). Real-Time and Correlative Imaging of Localised Corrosion Events by High-Speed Atomic Force Microscopy. Microsc. Microanal..

[141] Wang H., Sharma S., Pailleret A., Brown B., Nešić S. (2022). Investigation of Corrosion Inhibitor Adsorption on Mica and Mild Steel Using Electrochemical Atomic Force Microscopy and Molecular Simulations. Corrosion.

[142] Shen J., Lai T., Yin Z., Chen Y., Wang K., Yan H., Song H., Liu R., Luo C., Hu Z. (2024). In-Situ AFM and Quasi-in-Situ Studies for Localized Corrosion in Mg-9Al-1Fe-(Gd) Alloys under 3.5 Wt.% NaCl Environment. J. Magnes. Alloys.

[143] Pura J.L., Salvo-Comino C., García-Cabezón C., Rodríguez-Méndez M.L. (2023). Concurrent Study of the Electrochemical Response and the Surface Alterations of Silver Nanowire Modified Electrodes by Means of EC-AFM. The Role of Electrode/Nanomaterial Interaction. Surf. Interfaces.

[144] To-A-Ran W., Mastoi N.R., Ha C.Y., Song Y.J., Kim Y.-J. (2024). Kelvin Probe Force Microscopy and Electrochemical Atomic Force Microscopy Investigations of Lithium Nucleation and Growth: Influence of the Electrode Surface Potential. J. Phys. Chem. Lett..

[145] Thaman H.L., Li M., Rose J.A., Narasimhan S., Xu X., Yeh C.-N., Jin N., Akbashev A., Davidoff I., Bazant M.Z. (2025). Two-Stage Growth of Solid Electrolyte Interphase on Copper: Imaging and Quantification by *Operando* Atomic Force Microscopy. ACS Nano.

[146] Malladi S.R.K., Ummethala G., Jada R., Dutta-Gupta S., Park J., Tavabi A., Basak S., Hooley R., Sun H., Pérez-Garza H.H. (2023). Real-Time Visualisation of Organic Crystal Growth Dynamics during Liquid Reagent Mixing by Liquid Cell Transmission Electron Microscopy. preprint.

[147] Koo K., Seo J.H., Lee J., Lee S., Kwon J.-H. (2024). Investigating Charge-Induced Transformations of Metal Nanoparticles in a Radically-Inert Liquid: A Liquid-Cell TEM Study. Nanomaterials.

[148] Lee S., Schneider N.M., Tan S.F., Ross F.M. (2023). Temperature Dependent Nanochemistry and Growth Kinetics Using Liquid Cell Transmission Electron Microscopy. ACS Nano.

[149] Tarnawski T., Parlińska-Wojtan M. (2024). Opportunities and Obstacles in LCTEM Nanoimaging—A Review. Chem. Methods.

[150] Yang Y., Shao Y.-T., Lu X., Yang Y., Ko H.-Y., DiStasio R.A., DiSalvo F.J., Muller D.A., Abruña H.D. (2022). Elucidating Cathodic Corrosion Mechanisms with Operando Electrochemical Transmission Electron Microscopy. J. Am. Chem. Soc..

[151] Yang Y., Shao Y.-T., Jin J., Feijóo J., Roh I., Louisia S., Yu S., Guzman M.V.F., Chen C., Muller D.A. (2023). *Operando* Electrochemical Liquid-Cell Scanning Transmission Electron Microscopy (EC-STEM) Studies of Evolving Cu Nanocatalysts for CO_2_ Electroreduction. ACS Sustain. Chem. Eng..

[152] Dachraoui W., Pauer R., Battaglia C., Erni R. (2023). Operando Electrochemical Liquid Cell Scanning Transmission Electron Microscopy Investigation of the Growth and Evolution of the Mosaic Solid Electrolyte Interphase for Lithium-Ion Batteries. ACS Nano.

[153] Majeed M.N., Yousif Q.A., Bedair M.A. (2022). Study of the Corrosion of Nickel–Chromium Alloy in an Acidic Solution Protected by Nickel Nanoparticles. ACS Omega.

[154] Gattu V.K., Obregon J., Ebert W.L., Indacochea J.E. (2024). Effects of Open Circuit Immersion and Vertex Potential on Potentiodynamic Polarization Scans of Metallic Biomaterials. npj Mater. Degrad..

[155] Ott N., Fillon A.T., Renk O., Kremmer T., Pogatscher S., Suter T., Schmutz P. (2024). Local Electrochemical Characterization of Active Mg-Fe Materials—From Pure Mg to Mg50-Fe Composites. arXiv.

[156] Gerengi H., Cabrini M., Solomon M.M., Kaya E. (2022). Understanding the Corrosion Behavior of the AZ91D Alloy in Simulated Body Fluid through the Use of Dynamic EIS. ACS Omega.

[157] Branzoi F., Băran A., Mihai M.A., Praschiv A. (2025). Anticorrosive Effect of New Polymer Composite Coatings on Carbon Steel in Aggressive Environments by Electrochemical Procedures. Coatings.

[158] Akbari Y.H.A., Rostami M., Sari M.G., Ramezanzadeh B. (2024). pH-Responsive Anti-Corrosion Activity of Gallic Acid-Intercalated MgAl LDH in Acidic, Neutral, and Alkaline Environments. Mater. Today Commun..

[159] Haddadi S.A., Hu S., Ghaderi S., Ghanbari A., Ahmadipour M., Pung S.-Y., Li S., Feilizadeh M., Arjmand M. (2021). Amino-Functionalized MXene Nanosheets Doped with Ce(III) as Potent Nanocontainers toward Self-Healing Epoxy Nanocomposite Coating for Corrosion Protection of Mild Steel. ACS Appl. Mater. Interfaces.

[160] Abdi J., Izadi M., Bozorg M. (2022). Improvement of Anti-Corrosion Performance of an Epoxy Coating Using Hybrid UiO-66-NH2/Carbon Nanotubes Nanocomposite. Sci. Rep..

[161] Khorgami G., Arash Haddadi S., Okati M., Mekonnen T.H., Ramezanzadeh B. (2024). In Situ-Polymerized and Nano-Hybridized Ti3C2-MXene with PDA and Zn-MOF Carrying Phosphate/Glutamate Molecules; toward the Development of pH-Stimuli Smart Anti-Corrosion Coating. Chem. Eng. J..

[162] An H., Jiang C., Yin X., Liu K., Liang S., Wang X., Xiao J., Zhao X., Sun Z. (2025). Polyaniline/TiO_2_/MXene Ternary Composites for Enhancing Corrosion Resistance of Waterborne Epoxy Coatings. ACS Appl. Nano Mater..

[163] Gong J., Wei H., Hao P., Li S., Zhao X., Tang Y., Zuo Y. (2024). Study on the Influence of Metal Substrates on Protective Performance of the Coating by EIS. Materials.

[164] Wang Y., Howard C.B., Xu F., Salvato D., Bawane K.K., Murray D.J., Frazer D.M., Anderson S.T., Yao T., Yeo S. (2024). Microstructural and Micromechanical Characterization of Cr Diffusion Barrier in ATR Irradiated U-10Zr Metallic Fuel. J. Nucl. Mater..

[165] Wang B., Bai T., Wang W., Zhang H. (2024). Mechanical Properties of Silicon Nitride in Different Morphologies: In Situ Experimental Analysis of Bulk and Whisker Structures. Materials.

[166] Wang A., Song Y., Liang T., Ma D., Wang J., Xie J. (2024). Interfacial Microstructure of Dual-Scale SiCp/A356 Composites Investigated by in Situ TEM Tensile Testing. J. Alloys Compd..

[167] Loginov P.A., Zaitsev A.A., Sidorenko D.A., Eganova E.M., Levashov E.A. (2025). Interfacial Interaction and Evaluation of Bonding Strength between Diamond and CoCrFeNi(Cu,Ti) High-Entropy Alloys. Diam. Relat. Mater..

[168] Vermeij T., Sharma A., Steinbach D., Lou J., Michler J., Maeder X. (2025). In Situ Transmission Kikuchi Diffraction Tensile Testing. Scr. Mater..

[169] Nutor R.K., Azeemullah M., Cao Q.P., Wang X.D., Zhang D.X., Jiang J.Z. (2021). Microstructure and Properties of a Co-Free Fe_50_Mn_27_Ni_10_Cr_13_ High Entropy Alloy. J. Alloys Compd..

[170] Zhang Y., Wu H., Yu X., Tang D., Yuan R., Sun H. (2021). Microstructural Evolution and Strengthening Mechanisms in CrxMnFeNi High-Entropy Alloy. J. Mater. Res. Technol..

[171] Vermeij T., Verstijnen J.A.C., Ramirez Y Cantador T.J.J., Blaysat B., Neggers J., Hoefnagels J.P.M. (2022). A Nanomechanical Testing Framework Yielding Front&Rear-Sided, High-Resolution, Microstructure-Correlated SEM-DIC Strain Fields. Exp. Mech..

[172] Zhou J., Zhu M., Han Y., Zhou X., Wang S., Chen J., Wu H., Hou Y., Lu Y. (2024). Direct Measurement of Tensile Mechanical Properties of Few-Layer Hexagonal Boron Nitride (h-BN). J. Appl. Phys..

[173] Yang C., Liu Y., Wang J., Wu D., Liu L., Su Z., Xiong Y. (2023). Application of Nanoindentation Technique in Mechanical Characterization of Organic Matter in Shale: Attentive Issues, Test Protocol, and Technological Prospect. Gas Sci. Eng..

[174] Vanpée S., Nysten B., Chevalier J., Pardoen T. (2025). Nanoindentation Analysis of Transcrystalline Layers in Model Carbon Fiber-Reinforced PEEK Composite. Polym. Test..

[175] Saito I., Sheridan R.J., Zauscher S., Brinson L.C. (2025). Pushing AFM to the Boundaries: Interphase Mechanical Property Measurements near a Rigid Body. Macromolecules.

[176] Enrriques A.E., Howard S., Timsina R., Khadka N.K., Hoover A.N., Ray A.E., Ding L., Onwumelu C., Nordeng S., Mainali L. (2022). Atomic Force Microscopy Cantilever-Based Nanoindentation: Mechanical Property Measurements at the Nanoscale in Air and Fluid. J. Vis. Exp..

[177] Kontomaris S.V., Malamou A., Stylianou A. (2025). Simplifying Data Processing in AFM Nanoindentation Experiments on Thin Samples. Eng.

[178] Oliver W.C., Pharr G.M. (1992). An Improved Technique for Determining Hardness and Elastic Modulus Using Load and Displacement Sensing Indentation Experiments. J. Mater. Res..

[179] Wang J., Dziadkowiec J., Liu Y., Jiang W., Zheng Y., Xiong Y., Peng P., Renard F. (2024). Combining Atomic Force Microscopy and Nanoindentation Helps Characterizing In-Situ Mechanical Properties of Organic Matter in Shale. Int. J. Coal Geol..

[180] Pacheco L.R.L., Ferreira J.P.S., Parente M.P.L. (2024). Deep Learning Regressors of Surface Properties from Atomic Force Microscopy Nanoindentations. Appl. Sci..

[181] Yang X., Xi Y., He C., Chen H., Zhang X., Tu S. (2022). Chemical Short-Range Order Strengthening Mechanism in CoCrNi Medium-Entropy Alloy under Nanoindentation. Scr. Mater..

[182] Tong Y., Zhang H., Huang H., Yang L., Hu Y., Liang X., Hua M., Zhang J. (2021). Strengthening Mechanism of CoCrNiMox High Entropy Alloys by High-Throughput Nanoindentation Mapping Technique. Intermetallics.

[183] Okoro A.M., Lephuthing S.S., Rasiwela L., Olubambi P.A. (2021). Nondestructive Measurement of the Mechanical Properties of Graphene Nanoplatelets Reinforced Nickel Aluminium Bronze Composites. Heliyon.

[184] Dhal A., Agrawal P., Haridas R.S., Gaddam S., Sharma A., Parganiha D., Mishra R.S., Kawanaka H., Matsushita S., Yasuda Y. (2023). High-Throughput Investigation of Multiscale Deformation Mechanism in Additively Manufactured Ni Superalloy. Metals.

[185] Alroy R.J., Seekala H., Phani P.S., Sivakumar G. (2024). Role of High-Speed Nanoindentation Mapping to Assess the Structure-Performance Correlation of HVAF-Sprayed Cr_3_C_2_-25NiCr Coating. Surf. Coat. Technol..

[186] Singh P.P., Ranganathan R. (2022). Tensile and Viscoelastic Behavior in Nacre-Inspired Nanocomposites: A Coarse-Grained Molecular Dynamics Study. Nanomaterials.

[187] Noyel J.-P., Hajjar A., Debastiani R., Antouly K., Atli A. (2024). Impact of Viscoelasticity on the Stiffness of Polymer Nanocomposites: Insights from Experimental and Micromechanical Model Approaches. Polymer.

[188] Yazdanparast R., Rafiee R. (2024). A 3D Viscoelastic–Viscoplastic Behavior of Carbon Nanotube-reinforced Polymers: Constitutive Model and Experimental Characterization. Polym. Compos..

[189] Sánchez-Rodríguez C., Avilés M.-D., Pamies R., Carrión-Vilches F.-J., Sanes J., Bermúdez M.-D. (2021). Extruded PLA Nanocomposites Modified by Graphene Oxide and Ionic Liquid. Polymers.

[190] Madarvoni S., Ps Rama S. (2022). Dynamic Mechanical Behaviour of Graphene, Hexagonal Boron Nitride Reinforced Carbon-Kevlar, Hybrid Fabric-Based Epoxy Nanocomposites. Polym. Polym. Compos..

[191] Shivakumar H., Gurumurthy G.D., Yogananda G.S., Mahesh T.S., Bommegowda K.B. (2024). Dynamic Mechanical Properties of Graphene and Carbon Fabric-reinforced Epoxy Nanocomposites. Polym. Compos..

[192] Mat Yazik M.H., Sultan M.T.H., Jawaid M., Abu Talib A.R., Mazlan N., Md Shah A.U., Safri S.N.A. (2021). Effect of Nanofiller Content on Dynamic Mechanical and Thermal Properties of Multi-Walled Carbon Nanotube and Montmorillonite Nanoclay Filler Hybrid Shape Memory Epoxy Composites. Polymers.

[193] Ain Q.U., Wani M.F., Sehgal R., Singh M.K. (2024). Mechanical and Viscoelastic Characterization of Al_2_O_3_ Based Polymer Nanocomposites: An Experimental and Molecular Dynamics Simulation Approach. Comput. Mater. Sci..

[194] Yao C.-W., Lian I. (2025). Nanoscale Insights into the Mechanical and Tribological Properties of a Nanocomposite Coating. Nanomaterials.

[195] Yan C., Bor B., Plunkett A., Domènech B., Maier-Kiener V., Giuntini D. (2023). Nanoindentation Creep of Supercrystalline Nanocomposites. Mater. Des..

[196] Yang L., Chen Y., Miller J., Weber W.J., Bei H., Zhang Y. (2022). Deformation Mechanisms in Single Crystal Ni-Based Concentrated Solid Solution Alloys by Nanoindentation. Mater. Sci. Eng. A.

[197] Huang Y., Zhou C., Chen K., Yang Y., Xiong J., Yang J., Guo Y., Mao G., Yang L., Nie F. (2023). Nanoindentation Size Effects of Mechanical and Creep Performance in Ni-Based Superalloy. Mater. Sci. Technol..

[198] Dai Y., Zan Z., Zhao L., Qin F. (2024). Nanoindentation Elastoplastic and Creep Behaviors of Sintered Nano-Silver Doped with Nickel-Modified Multiwall Carbon Nanotube Filler. J. Electron. Mater..

[199] Yao C.-W., Lian I., Zhou J., Bernazzani P., Jao M. (2025). The Elevated-Temperature Nano-Mechanical Properties of a PDMS–Silica-Based Superhydrophobic Nanocomposite Coating. Nanomaterials.

[200] Darnbrough E., Aspinall J., Pasta M., Armstrong D.E.J. (2023). Elastic and Plastic Mechanical Properties of Lithium Measured by Nanoindentation. Mater. Des..

[201] Li J., Wang C., Liu J., Dong X., Zhao J., Chen Y. (2024). Simulation and Experimental Study on Stress Relaxation Response of Polycrystalline γ-TiAl Alloy under Nanoindentation Based on Molecular Dynamics. Micromachines.

[202] Holz H., Merle B. (2023). Novel Nanoindentation Strain Rate Sweep Method for Continuously Investigating the Strain Rate Sensitivity of Materials at the Nanoscale. Mater. Des..

[203] Pomes S., Adachi N., Wakeda M., Ohmura T. (2023). Probing Pre-Serration Deformation in Zr-Based Bulk Metallic Glass via Nanoindentation Testing. Scr. Mater..

[204] Zhang Y., Li J., Hu Y., Ding S., Wu W., Xia R. (2024). Fatigue Responses of Metallic Glass-based Stochastic Network Nanomaterial: Superior Strain-hardening Ability. Fatigue Fract. Eng. Mater. Struct..

[205] Schmahl M., Müller C., Meinke R., Alcantara E.G.A., Hangen U.D., Fleck C. (2023). Cyclic Nanoindentation for Local High Cycle Fatigue Investigations: A Methodological Approach Accounting for Thermal Drift. Adv. Eng. Mater..

[206] Huang W., Yan J. (2023). Towards Understanding the Mechanism of Vibration-Assisted Cutting of Monocrystalline Silicon by Cyclic Nanoindentation. J. Mater. Process. Technol..

[207] Owhal A., Belwanshi V., Roy T., Goel S. (2024). Strain Softening Observed during Nanoindentation of Equimolar-Ratio Co–Mn–Fe–Cr–Ni High Entropy Alloy. J. Micromanufacturing.

[208] Luo R., Wang B., Wang Q., Yu J., Gao Z. (2024). Cyclic Degradation Mechanisms of Al_0.3_CoCrFeNi High-Entropy Alloy under Different Loading Rates. Mater. Today Commun..

[209] Kovalev A.I., Vakhrushev V.O., Beake B.D., Konovalov E.P., Wainstein D.L., Dmitrievskii S.A., Fox-Rabinovich G.S., Veldhuis S. (2022). Damage Accumulation Phenomena in Multilayer (TiAlCrSiY)N/(TiAlCr)N, Monolayer (TiAlCrSiY)N Coatings and Silicon upon Deformation by Cyclic Nanoindentation. Nanomaterials.

[210] Hübler D., Winkler K., Riedel R., Kamrani S., Fleck C. (2022). Cyclic Deformation Behavior of Mg–SiC Nanocomposites on the Macroscale and Nanoscale. Fatigue Fract. Eng. Mater. Struct..

[211] Jha M., Shimpi N.G. (2022). Mechanical Response of Silver/Polyvinyl Alcohol Thin Film: From One-Step and Cyclic Nanoindentation. Adv. Ind. Eng. Polym. Res..

[212] Sowa S., Kacprzyńska-Gołacka J., Smolik J., Wieciński P. (2025). Mechanical Properties of Cu+CuO Coatings Determined by Nanoindentation and Laugier Model. Materials.

[213] Smolik J., Sowa S., Kacprzyńska-Gołacka J., Piasek A. (2022). Evaluation of the Fracture Toughness KIc for Selected Magnetron Sputtering Coatings by Using the Laugier Model. Materials.

[214] Gautham S., Sasmal S. (2022). Nano-Scale Fracture Toughness of Fly Ash Incorporated Hydrating Cementitious Composites Using Experimental Nanoindentation Technique. Theor. Appl. Fract. Mech..

[215] Günen A., Makuch N., Altınay Y., Çarboğa C., Dal S., Karaca Y. (2022). Determination of Fracture Toughness of Boride Layers Grown on Co_1.21_Cr_1.82_Fe_1.44_Mn_1.32_Ni_1.12_Al_0.08_B_0.01_ High Entropy Alloy by Nanoindentation. Ceram. Int..

[216] Lapitskaya V.A., Kuznetsova T.A., Chizhik S.A., Rogachev A.A. (2024). Determination of Fracture Toughness of the Thin Diamond-like Coatings by Nanoindentation. Proc. Natl. Acad. Sci. Belarus Phys. Tech. Ser..

[217] Zhang M., Zhang G., Peng Y. (2024). Characterizing the Micro-Fracture in Quasi-Brittle Rock Using Nanoindentation. Eng. Fract. Mech..

[218] Sakhaee-Pour A. (2025). Deep Learning for Characterizing Fracture Toughness from the Nanoindentation Image of a Complex Heterogeneous Medium. Theor. Appl. Fract. Mech..

[219] Beake B.D., Vishnyakov V.M., Zhang H., Goodes S.R., Rahmati A.T. (2025). Statistically Distributed Nano-Scratch Testing—A Novel Method for Simulating Abrasive Wear. Wear.

[220] Dewangan S.K., Cheenepalli N., Lee H., Ahn B. (2024). Microstructure and Nanoscratch Behavior of Spark-Plasma-Sintered Ti-V-Al-Nb-Hf High-Entropy Alloy. J. Mater. Res. Technol..

[221] Zhang L., Gain A.K., Li Z. (2025). Exploring the Deformation Mechanisms and Mechanical Properties of Fused Silica through Nanoindentation and Ramp-Nanoscratching. Wear.

[222] Brüssel F., Huang W., Yan J. (2024). Investigation of Failure Modes and Material Structural Responses of Nanographite Coatings on Single-Crystal Silicon by Nanoscratching. Tribol. Int..

[223] Li Z., Li Y., Zhang L. (2024). On the Deformation Mechanism and Dislocations Evolution in Monocrystalline Silicon under Ramp Nanoscratching. Tribol. Int..

[224] Beake B.D., Vishnyakov V.M., Goodes S.R., Rahmati A.T. (2024). Statistically Distributed Nano-Scratch Testing of AlFeMnNb, AlFeMnNi, and TiN/Si3N4 Thin Films on Silicon. J. Vac. Sci. Technol. A.

[225] Wilsnack E., Zawischa M., Makowski S., Zimmermann M., Leyens C. (2024). Low Cycle Fatigue of Doped Tetrahedral Amorphous Carbon Coatings by Repetitive Micro Scratch Tests. Surf. Coat. Technol..

[226] Wang W., Tian Y., Zhang Z., Lu Z., Wang F., Zhang D. (2024). Investigation of Cutting Depth and Contact Area in Nanoindenter Scratching. Precis. Eng..

[227] Liu T., Wu H., Liu Y., Huang H. (2024). The Effects of the Angle between the Indenter Edge and the Scratch Direction on the Scratch Characteristics of Ti–6Al–4V Alloy. Wear.

[228] Huang N., Zhou P., Goel S. (2023). Microscopic Stress Analysis of Nanoscratch Induced Sub-Surface Defects in a Single-Crystal Silicon Wafer. Precis. Eng..

[229] Hu J., Zeng Q. (2022). Friction and Wear in Nanoscratching of Single Crystals: Effect of Adhesion and Plasticity. Nanomaterials.

[230] Bennewitz R. (2024). Friction Force Microscopy. Fundamentals of Friction and Wear on the Nanoscale.

[231] Dašić M., Almog R., Agmon L., Yehezkel S., Halfin T., Jopp J., Ya’akobovitz A., Berkovich R., Stanković I. (2024). Role of Trapped Molecules at Sliding Contacts in Lattice-Resolved Friction. ACS Appl. Mater. Interfaces.

[232] Miyata R., Inoue S., Nikaido K., Nakajima K., Hasegawa T. (2024). Friction Force Mapping of Molecular Ordering and Mesoscopic Phase Transformations in Layered-Crystalline Organic Semiconductor Films. ACS Appl. Mater. Interfaces.

[233] Park S., Nguyen P.L., Vlassiouk I.V., Choi M., Kim S., Lee J., Kim S. (2024). Unveiling the Mechanism of Surface Corrugation Formation on a Quasi Free-Standing Bi-Layer Graphene via Experimental and Modeling Investigations. Appl. Surf. Sci..

[234] Ma C., Li Y., Zhou C., Chen Y., Gnecco E., Chu J. (2024). Shear Anisotropy Domains on Graphene Revealed by In-Plane Elastic Imaging. ACS Nano.

[235] Huynh N.-P., Kim H.-J., Chung K.-H. (2026). Long-Term Wear Characteristics of Single-Layer h-BN, MoS 2, and Graphene. Tribol. Int..

[236] Xu C., Egberts P. (2024). Triboelectrification and Unique Frictional Characteristics of Germanium-Based Nanofilms. Small.

[237] Aksaray G., Mert M.E., Doğru Mert B. (2025). DFT Approach in Corrosion Research. Osman. Korkut Ata Üniversitesi Fen Bilim. Enstitüsü Derg..

[238] Chen D., Zhou W., Ji Y., Dong C. (2025). Applications of Density Functional Theory to Corrosion and Corrosion Prevention of Metals: A Review. Mater. Genome Eng. Adv..

[239] Liu X., Gao K., Chen P., Yin L., Yang J. (2025). Multiscale Simulation of Nanowear-Resistant Coatings. Materials.

[240] Xu D., Pei Z., Yang X., Li Q., Zhang F., Zhu R., Cheng X., Ma L. (2023). A Review of Trends in Corrosion-Resistant Structural Steels Research—From Theoretical Simulation to Data-Driven Directions. Materials.

[241] Zeng C., Neils A., Lesko J., Post N. (2024). Machine Learning Accelerated Discovery of Corrosion-Resistant High-Entropy Alloys. Comput. Mater. Sci..

[242] Wang Y., Xie T., Tang Q., Wang M., Ying T., Zhu H., Zeng X. (2024). High-Throughput Calculations Combining Machine Learning to Investigate the Corrosion Properties of Binary Mg Alloys. J. Magnes. Alloys.

[243] Hasan S., Berkeley G., Polifrone K., Xu W. (2022). An Atomistic Study of Deformation Mechanisms in Metal Matrix Nanocomposite Materials. Mater. Today Commun..

[244] Hoang K.-Q., Kadapa C., Chakraverty S. (2021). Multiscale Modeling for the Statics of Nanostructures. Nano Scaled Structural Problems.

[245] Ji J., Jin Y., Hua A., Zhu C., Zhao J. (2023). Multiscale Theories and Applications: From Microstructure Design to Macroscopic Assessment for Carbon Nanotubes Networks. Chin. J. Mech. Eng..

[246] Khoei A.R., Seddighian M.R., Sameti A.R. (2024). Machine Learning-Based Multiscale Framework for Mechanical Behavior of Nano-Crystalline Structures. Int. J. Mech. Sci..

[247] Winetrout J.J., Li Z., Zhao Q., Gaber L., Unnikrishnan V., Varshney V., Xu Y., Wang Y., Heinz H. (2025). Prediction of Carbon Nanostructure Mechanical Properties and the Role of Defects Using Machine Learning. Proc. Natl. Acad. Sci. USA.

[248] Champa-Bujaico E., Díez-Pascual A.M., Lomas Redondo A., Garcia-Diaz P. (2024). Optimization of Mechanical Properties of Multiscale Hybrid Polymer Nanocomposites: A Combination of Experimental and Machine Learning Techniques. Compos. Part B Eng..

[249] Pasha M.B., Rao R.N., Ismail S., Gupta M., Prasad P.S. (2024). Tribo-Informatics Approach to Predict Wear and Friction Coefficient of Mg/Si3N4 Composites Using Machine Learning Techniques. Tribol. Int..

[250] Talapatra A., Datta D. (2024). Experimental and Molecular Dynamics Simulation Based Investigation to Understand Tribological Performance of Graphene Reinforced Thermoplastic Polyurethane (Gr/TPU) Nanocomposites. Tribol. Int..

[251] Prasad M.B., Sahu S.K. (2025). Taguchi and Machine Learning Integration for Tribological Analysis of Polyurethane/Nanodiamond Nanocomposites. Proc. Inst. Mech. Eng. Part J J. Eng. Tribol..

[252] Aherwar A., Ahirwar A., Pathak V.K. (2025). Dry Sliding Tribological Characteristics Evaluation and Prediction of TiB2-CDA/Al6061 Hybrid Composites Exercising Machine Learning Methods. Sci. Rep..

[253] Aherwar A., Ahirwar A., Pathak V.K. (2025). Triboinformatic Analysis and Prediction of B4C and Granite Powder Filled Al 6082 Composites Using Machine Learning Regression Models. Sci. Rep..

[254] Kolev M., Drenchev L., Petkov V. (2023). Fabrication, Experimental Investigation and Prediction of Wear Behavior of Open-Cell AlSi10Mg-SiC Composite Materials. Metals.

[255] Jin S., Qiao H., Zhao J. (2025). Temperature-dependent Tribological and Interfacial Properties of Perfluoroelastomer Nanocomposites Modified with Graphene Nanosheets Functionalized through Molecular Dynamics Simulations. Polym. Compos..

[256] Etsuyankpa M.B., Hassan I., Musa S.T., Mathew J.T., Shaba E.Y., Andrew A., Muhammad A.I., Muhammad K.T., Jibrin N.A., Abubakar M.K. (2024). Comprehensive Review of Recent Advances in Nanoparticle-Based Corrosion Inhibition Approaches. J. Appl. Sci. Environ. Manag..

[257] Galluzzi M., Lancia M., Zheng C., Re V., Castelvetro V., Guo S., Viaroli S. (2025). Atomic Force Microscopy (AFM) Nanomechanical Characterization of Micro- and Nanoplastics to Support Environmental Investigations in Groundwater. Emerg. Contam..

[258] Doniger W.H., Couet A., Sridharan K. (2022). Potentiodynamic Polarization of Pure Metals and Alloys in Molten LiF-NaF-KF (FLiNaK) Using the K/K ^+^ Dynamic Reference Electrode. J. Electrochem. Soc..

[259] Zhao Z., Zhou M., Zhao W., Hu J., Fu H. (2022). Anti-Corrosion Epoxy/Modified Graphene Oxide/Glass Fiber Composite Coating with Dual Physical Barrier Network. Prog. Org. Coat..

[260] Al-Gorair A.S., Saleh M.G.A., Alotaibi M.T., Al-Juaid S.S., Abdallah M., Wanees S.A.E. (2023). Potentiometric and Polarization Studies on the Oxide Film Repair and Retardation of Pitting Corrosion on Indium in an Alkaline Aqueous Solution Utilizing Some Triazole Compounds. Inorg. Chem. Commun..

[261] Shanbaraki Z.D., Azadi M., Hafazeh A. (2024). Electrochemical Characteristics of Nano-Structure TiCN Coatings on the Tool Steel Deposited by PACVD in Various Solutions. Results Chem..

[262] Batakliev T., Ivanov E., Georgiev V., Angelov V., Ahuir-Torres J.I., Harvey D.M., Kotsilkova R. (2024). New Insights in the Nanomechanical Study of Carbon-Containing Nanocomposite Materials Based on High-Density Polyethylene. Appl. Sci..

[263] Bendaoued A., Salhi R. (2025). A Comprehensive Analysis of the Structural, Textural, and Nanomechanical Properties of Sol–Gel Synthesized TiO_2_, Al_2_O_3_, and SiO_2_ Nanoparticles. Euro Mediterr. J. Environ. Integr..

[264] Singh V., Sharma R.K., Sehgal R. (2024). An Experimental Investigation on Nanomechanical and Nanotribological Behavior of Tantalum Nitride Coating Deposited on Ti6Al7Nb Alloy. Tribol. Int..

[265] Iteney H., Cornelius T.W., Thomas O., Amodeo J. (2024). Influence of Surface Roughness on the Deformation of Gold Nanoparticles under Compression. Acta Mater..

[266] Wang J., Gong L., Xi S., Li C., Su Y., Yang L. (2024). Synergistic Effect of Interface and Agglomeration on Young’s Modulus of Graphene-Polymer Nanocomposites. Int. J. Solids Struct..

[267] Mazaheri M., Payandehpeyman J., Hedayatian M. (2024). Agglomeration and Interphase-Influenced Effective Elastic Properties of Metal/Graphene Nanocomposites: A Developed Mean-Field Model. Compos. Struct..

[268] Rościszewska M., Shimabukuro M., Ronowska A., Mielewczyk-Gryń A., Zieliński A., Hanawa T. (2024). Enhanced Bioactivity and Mechanical Properties of Silicon-Infused Titanium Oxide Coatings Formed by Micro-Arc Oxidation on Selective Laser Melted Ti13Nb13Zr Alloy. Ceram. Int..

[269] Huang Y., Nie M., Li B., Wu B., Zheng A., Yin K., Sun L. (2023). Real-Time Quantitative Electromechanical Characterization of Nanomaterials Based on Integrated MEMS Device. IEEE Sens. J..

[270] Mishra M.K., Mahur P., Manimunda P., Mishra K. (2023). Recent Advances in Nanomechanical Measurements and Their Application for Pharmaceutical Crystals. Mol. Pharm..

[271] Kiener D., Wurmshuber M., Alfreider M., Schaffar G.J.K., Maier-Kiener V. (2023). Recent Advances in Nanomechanical and in Situ Testing Techniques: Towards Extreme Conditions. Curr. Opin. Solid State Mater. Sci..

[272] Dash R., Bhattacharyya K., Bhattacharyya A.S. (2023). Stress Distribution Variations during Nanoindentation Failure of Hard Coatings on Silicon Substrates. Nanotechnol. Precis. Eng..

[273] Zak S. (2024). Controlling Strain Localization in Thin Films with Nanoindenter Tip Sharpness. Sci. Rep..

[274] Kontomaris S.V., Stylianou A., Chliveros G., Malamou A. (2023). A New Elementary Method for Determining the Tip Radius and Young’s Modulus in AFM Spherical Indentations. Micromachines.

[275] Liang T., Yu Q., Yin Z., Chen S., Liu Y., Yang Y., Lou H., Shen B., Zeng Z., Zeng Q. (2022). Spatial Resolution Limit for Nanoindentation Mapping on Metallic Glasses. Materials.

[276] Liang K., Gao K., Cai W. (2022). The Study on the Substrate Effect in the Nanoindentation Experiment of the Hybrid Material. Adv. Civ. Eng..

[277] Liu Y., Zhang J., Guo Q. (2022). Nanoindentation Study on Helium-Irradiated Graphene–Aluminum Composite: Indentation Size Effect, Creep Behavior, and Their Implications for Coating–Substrate Systems. J. Mater. Res..

[278] Minnert C., Durst K. (2022). Nanoindentation Creep Testing: Advantages and Limitations of the Constant Contact Pressure Method. J. Mater. Res..

[279] Sokoli V., Kamnis S., Delibasis K., Georgatis E., Kiape S., Karantzalis A.E. (2024). The Advanced Assessment of Nanoindentation-Based Mechanical Properties of a Refractory MoTaNbWV High-Entropy Alloy: Metallurgical Considerations and Extensive Variable Correlation Analysis. Appl. Sci..

[280] Rahman M.S., Polycarpou A.A. (2022). Nanomechanical and Nanoscratch Behavior of Oxides Formed on Inconel 617 at 950 °C. J. Mater. Res..

